# NKG2D receptor activation drives primary graft dysfunction severity and poor lung transplantation outcomes

**DOI:** 10.1172/jci.insight.164603

**Published:** 2022-12-22

**Authors:** Daniel R. Calabrese, Tasha Tsao, Mélia Magnen, Colin Valet, Ying Gao, Beñat Mallavia, Jennifer J. Tian, Emily A. Aminian, Kristin M. Wang, Avishai Shemesh, Elman B. Punzalan, Aartik Sarma, Carolyn S. Calfee, Stephanie A. Christenson, Charles R. Langelier, Steven R. Hays, Jeffrey A. Golden, Lorriana E. Leard, Mary Ellen Kleinhenz, Nicholas A. Kolaitis, Rupal Shah, Aida Venado, Lewis L. Lanier, John R. Greenland, David M. Sayah, Abbas Ardehali, Jasleen Kukreja, S. Samuel Weigt, John A. Belperio, Jonathan P. Singer, Mark R. Looney

**Affiliations:** 1 Department of Medicine, San Francisco Veterans Affairs Medical Center, San Francisco, California, USA.; 2 Department of Medicine, UCSF, San Francisco, California, USA.; 3 Parker Institute for Cancer Immunotherapy, San Francisco, California, USA.; 4 Department of Medicine, UCLA, Los Angeles, California, USA.; 5 Department of Microbiology and Immunology and; 6 Department of Surgery, UCSF, San Francisco, California, USA.

**Keywords:** Immunology, Pulmonology, NK cells, Neutrophils, Organ transplantation

## Abstract

Clinical outcomes after lung transplantation, a life-saving therapy for patients with end-stage lung diseases, are limited by primary graft dysfunction (PGD). PGD is an early form of acute lung injury with no specific pharmacologic therapies. Here, we present a large multicenter study of plasma and bronchoalveolar lavage (BAL) samples collected on the first posttransplant day, a critical time for investigations of immune pathways related to PGD. We demonstrated that ligands for NKG2D receptors were increased in the BAL from participants who developed severe PGD and were associated with increased time to extubation, prolonged intensive care unit length of stay, and poor peak lung function. Neutrophil extracellular traps (NETs) were increased in PGD and correlated with BAL TNF-α and IFN-γ cytokines. Mechanistically, we found that airway epithelial cell NKG2D ligands were increased following hypoxic challenge. NK cell killing of hypoxic airway epithelial cells was abrogated with NKG2D receptor blockade, and TNF-α and IFN-γ provoked neutrophils to release NETs in culture. These data support an aberrant NK cell/neutrophil axis in human PGD pathogenesis. Early measurement of stress ligands and blockade of the NKG2D receptor hold promise for risk stratification and management of PGD.

## Graphical abstract

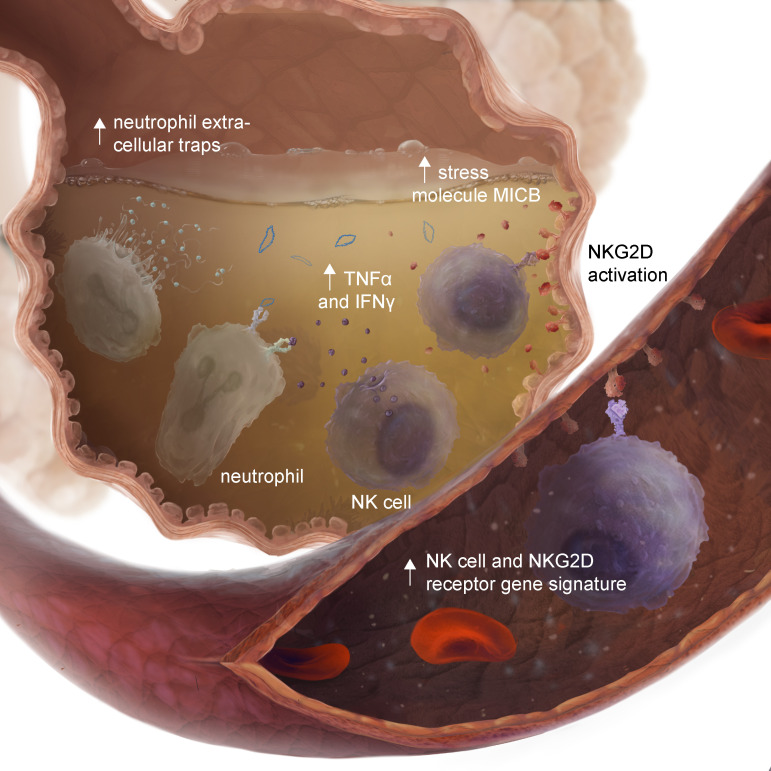

## Introduction

Lung transplantation is a life-prolonging therapy for patients with end-stage lung disease. However, the potential benefits of transplantation are limited by some of the highest risk for morbidity and mortality among solid organ transplants (1). Up to one-third of lung transplant recipients develop a severe form of primary graft dysfunction (PGD), the clinical manifestation of ischemia-reperfusion injury (2). PGD has no known medical therapies, accounts for half of the first-year mortality after lung transplantation, and is a major risk factor for chronic lung allograft dysfunction and impaired health-related quality of life (3). PGD shares pathophysiology with the acute respiratory distress syndrome (ARDS) and is characterized by biphasic inflammation. Activation of the pulmonary epithelium and endothelium launches a cascade of injury responses, including upregulation of adhesion markers and release of chemokines and damage-associated molecular patterns (4, 5). Before perfusion, donor TLR gene transcription and innate lymphoid cells have been implicated in PGD pathophysiology (6). Upon reperfusion, there is an influx of innate immune cells that ultimately results in compromised barrier function and protein-rich edema fluid within the alveolar airspaces (7). However, there is an incomplete understanding of how these events work in concert or an understanding of the early interaction between donor graft injury and the recipient innate immune responses.

Our previous work showed that recipient NK cells and neutrophils play critical roles in mouse models of PGD. NK cells are innate immune cells increasingly recognized as important in lung transplant outcomes (8–10). They identify altered- or missing-self through the integration of multiple activating and inhibiting signals downstream of a myriad of specific and targetable receptors (11, 12). NK cells are capable of direct cellular toxicity through granzyme or perforin release and FAS ligand interactions (13). They also mediate other innate immune cell recruitment as the largest source of IFN-γ in the lung (14). Recently, we demonstrated that NKG2D receptor–specific stress molecules are increased on pulmonary airway epithelial cells during a mouse model of PGD (15). NK cells mediated lung injury through recognition of these stress ligands by a receptor, NKG2D.

In parallel, we have shown that neutrophils accumulate in the PGD lung and impact lung injury via release of neutrophil extracellular traps (NETs) (16, 17). NETs are extrusions of DNA complexed with histones and granular proteins that are released in a regulated process to kill pathogens (18). NETs have also been implicated in tissue damage during autoimmune and sterile inflammatory processes (19). Our mouse models showed that depletion of NK cells in transplant recipients resulted in reduced neutrophil influx and injury (15). Other models of acute lung injury have implicated NK cells in neutrophil recruitment and function (20). However, the importance of these 2 innate immune cells in human PGD and their early interactions remains to be fully elucidated.

Here, we established a large, 2-center biorepository of bronchoalveolar lavage (BAL) and plasma samples obtained on the first day after lung transplant for the investigation of immune pathways that regulate PGD pathogenesis. Based on our previous preclinical work, we hypothesized that NKG2D stress ligands and neutrophils would be associated with the development of severe PGD. Clarifying these pathways and their interactions could identify novel treatment paradigms for PGD.

## Results

### Validation of PGD clinical risk factors.

Lung transplant recipient and donor clinical characteristics for the entire cohort are reported in Supplemental Table 1 (supplemental material available online with this article; https://doi.org/10.1172/jci.insight.164603DS1). Across our multicenter cohort, we observed that 21.1% (*N* = 105) of recipients developed severe PGD (Supplemental Table 2), defined as grade 3 PGD on postoperative day 2 (POD2) or POD3 (21, 22). We first sought to establish the validity of our cohort to identify the portability of inferences based on our biologic observations. As such, we evaluated the association between established clinical risk factors and severe PGD in a multivariate analysis, shown in Table 1. We identified that previously established major clinical risk factors (donor age, BMI, and pulmonary artery pressure) were associated with PGD within our multicenter cohort (2). However, we also identified the transplant indication of interstitial lung disease (ILD) as a potentially independent risk factor for severe PGD (*P* = 0.09). Notably, ILD other than idiopathic pulmonary fibrosis was independently associated with severe PGD (OR, 1.6; 95% CI, 1–2.6; *P* = 0.05). Concordant with prior reports, and highlighting an urgent unmet need, we observed nearly 19-fold higher odds of death in the first 90 days and 7.8-fold higher odds of death in the first year after transplant among recipients with severe PGD compared with those without PGD (Table 2).

### Gene scores identify distinct immune transcriptional responses.

**Figure 1:**
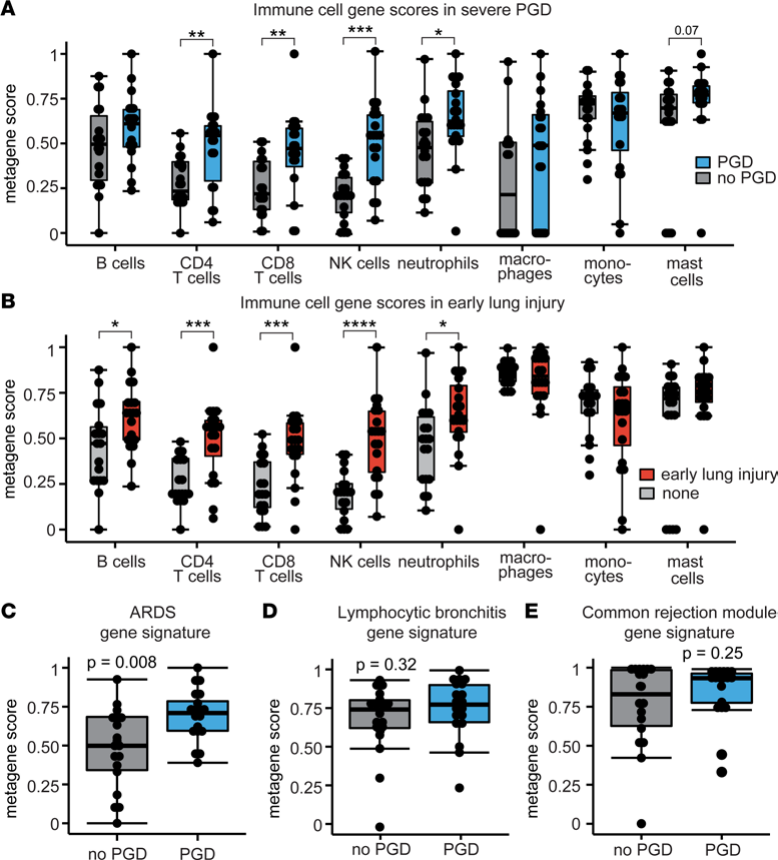
Immune cell and lung inflammation gene expression profiles. Bronchoalveolar lavage (BAL) RNA-Seq data are shown for participants with severe PGD (*N* = 19) and no PGD (*N* = 19). (**A**) Gene scores for immune cell subsets and PGD. (**B**) Immune cell gene scores and early inflammation, defined as grade 2 or 3 PGD on postoperative day 1. (**C**) An acute respiratory distress syndrome (ARDS) pathway gene score was constructed with differentially expressed genes from tracheal aspirates in a published study. (**D** and **E**) Other gene scores for lymphocytic bronchitis and common rejection module, were not increased among participants with severe PGD compared with participants without severe PGD. Data are displayed with box-and-whisker plots illustrating individual data points, bound by boxes at 25th and 75th percentiles, and with medians depicted with bisecting lines. Differences were assessed by Mann Whitney *U* test with *P* values shown or bracketed. *P < 0.05, **P < 0.01, ***P < 0.001, ****P < 0.0001.

We aimed to define the immune cell transcriptional landscape within the lung on POD1 after lung transplantation. We assessed BAL gene transcription differences in these POD1 samples between our participants who subsequently were graded on POD2 or POD3 with severe PGD (*N* = 19) or no PGD (*N* = 19). Supplemental Table 3 shows the composition of this group of participants, and Supplemental Figure 2 shows the results of the unsupervised genomic analyses. We used bulk RNA-Seq on cells recovered from the BAL to generate gene expression profiles for immune cell subsets based on previously published methodologies (23). We found that NK cell gene scores were increased 2.4-fold in participants who developed severe PGD (*P* = 0.0002) compared with participants without PGD (Figure 1A). Gene scores for neutrophils (1.3-fold, *P* = 0.01), CD4^+^ T cells (2.3-fold, *P* = 0.003), and CD8^+^ T cells (2.1-fold, *P* = 0.004) were also increased among participants who developed severe PGD relative to those who did not develop PGD. Gene scores for B cells, macrophages, monocytes, and mast cells were not different between the groups.

Since these samples were collected on POD1, we also assessed if immune cell gene scores were differentially expressed with high PGD grades early in the postoperative course. We termed this as early allograft injury, defined as grade 2 or 3 PGD on the day of collection (POD1). Similarly, we found NK cell transcripts had the most robust increase associated with early allograft injury (2.7-fold, *P* < 0.0001) compared with participants without early allograft injury (Figure 1B). Gene scores for neutrophils (1.3-fold, *P* = 0.02), CD4^+^ T cells (2.5-fold, *P* = 0.0002), and CD8^+^ T cells (2.5-fold, *P* = 0.0002) were also increased in participants with early allograft injury. The B cell gene score (1.3-fold, *P* = 0.04) was increased at this earlier time point among participants with early allograft injury (though not on POD3).

PGD shares clinical definitions and basic pathophysiology with ARDS. As such, we assembled a gene score using the top differentially expressed genes (absolute log_2_ fold-changes > 2 and FDR < 0.01) described in a study of tracheal aspirates among participants with ARDS and healthy controls (24). We found that ARDS genes were increased 1.7-fold (Figure 1C, *P* = 0.008) in participants who developed severe PGD compared with participants who did not develop PGD. Gene scores for lymphocytic bronchiolitis (Figure 1D, *P* = 0.32), another airway-centric inflammatory disease, and the common rejection module (Figure 1E, *P* = 0.25), a marker of acute cellular rejection, were not different in participants who developed PGD. These results suggest that common pathways are involved in pathobiology of PGD and ARDS and highlight that both innate and adaptive immune pathways are associated with the development of severe PGD. Since NK cell transcripts had the strongest association with development of severe PGD, we focused on these cells for the remainder of this study.

### NK cell, T cell, and NKG2D receptor pathways are associated with PGD.

**Figure 2:**
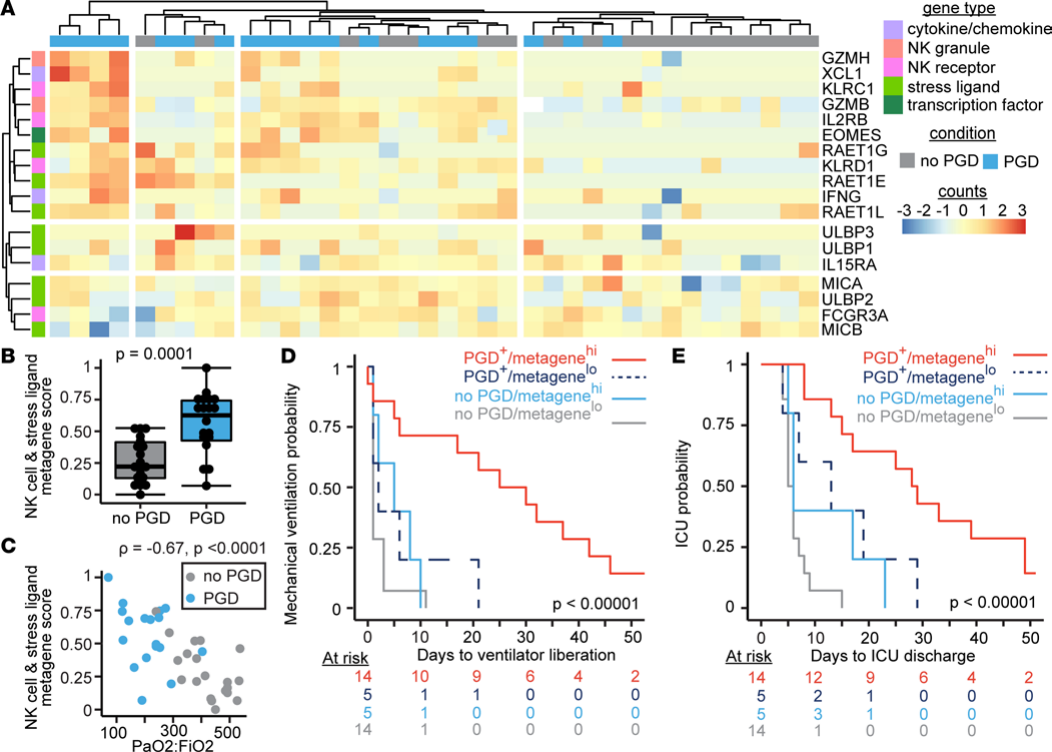
NK cell and stress ligand gene expression profiles are associated with PGD. A BAL NK cell and stress molecule gene score was generated from RNA-Seq obtained from 38 participants on the first day after lung transplantation (severe PGD, *N* = 19). (**A**) A heatmap displays normalized gene counts for the genes included in the score. (**B**) NK cell NKG2D receptor ligand stress molecule gene score in PGD. (**C**) Correlation plot of gene score plotted against arterial oxygen pressure relative to the fraction of inspired oxygen (PaO_2_/FiO_2_ ratio). (**D**) Kaplan-Meier plot of mechanical ventilation time stratified by NK cell and stress molecule gene scores and PGD. (**E**) Kaplan-Meier plot of ICU LOS stratified by NK cell and stress molecule gene scores and PGD. Heatmap data display row-normalized gene counts. Summary data are displayed with box-and-whisker plots illustrating individual data points, bound by boxes at 25th and 75th percentiles, and with medians depicted with bisecting lines. Individual *P* values are shown, and differences assessed with Mann Whitney *U* test (**B**), Spearman’s correlation (**C**), and log-rank test for Kaplan-Meier plots of mechanical ventilation (**D**) and ICU survival times (**E**).

We next examined if RNA transcripts common but not unique to NK cells (*GZMB*, *XCL1*, *KLRC1*, *IL2RB*, *EOMES*, *KLRD1*, and *FCGR3A*) and stress molecule RNA transcripts were increased in participants who developed severe PGD. Figure 2A shows the relative counts of genes comprising this NK and stress molecule pathway score (NK-stress). We found increased NK-stress gene scores in participants who developed severe PGD compared with those who did not develop PGD (Figure 2B, *P* = 0.0001). In ARDS, a measure of severity is the ratio of arterial oxygen relative to the fraction of inspired oxygen (PaO_2_/FiO_2_). Lower ratios (ratios < 300) indicate more severe disease. We found that the NK-stress gene score was negatively correlated with PaO_2_/FiO_2_ (Figure 2C, Spearman’s ρ = –0.67, *P* < 0.0001). These findings suggest that NK-stress transcripts measured within 24 hours of lung transplant are strongly associated with the severity of PGD on POD3.

We hypothesized that the presence of increased NK cell and stress molecule transcripts may capture information about the donor and recipient injury response and would be associated with poor early outcomes. Indeed, at any given time early after transplant, participants with higher than the median NK cell scores were almost 5-fold less likely to get extubated (hazard ratio [HR], 4.9; 95% CI, 2.1–11.6; *P* = 0.0003). After controlling for PGD status and baseline characteristics, an increased NK-stress gene score was independently associated with increased risk for the need for mechanical ventilation (Figure 2D) (HR, 3.2; 95% CI, 1.3–7.4; *P* = 0.009). We further evaluated if this gene score was associated with additional early transplant medical complexity and examined its association with intensive care unit (ICU) length of stay (LOS). Similarly, at any given time, participants with increased NK-stress gene scores were 4.2-fold less likely to transfer out of the ICU (95% CI, 2–9.1; *P* = 0.0002). After accounting for PGD status and baseline characteristics, an increased NK-stress gene score also remained an independent risk for prolonged ICU LOS (Figure 2E) (HR, 2.5; 95% CI, 1.1–6; *P* = 0.04). Since there is transcriptional overlap between CD8^+^ T cells and NK cells, we also assessed if CD8^+^ T cell gene scores were associated with mechanical ventilation or ICU LOS. Interestingly, we found no altered risk for prolonged mechanical ventilation or ICU LOS among participants with elevated CD8 gene scores (Supplemental Figure 3). These findings suggest that NK cells and stress molecules may be mechanistically associated with pulmonary PGD.

### NK cell growth factors and cytokines are increased in PGD.

We performed unsupervised analyses of 71 soluble analytes within the BAL of lung transplant recipients collected on the POD1. Supplemental Table 4 shows the demographics for this cohort. Supplemental Figure 4A shows the top 25% differentially expressed proteins in a heatmap segregated by PGD status. Supplemental Figure 4B displays BAL protein analytes that crossed our threshold of significance (negative log FDR–adjusted *P* < 1.2 and absolute log fold change > 0.3). Supplemental Figure 4 reveals that several cytokines previously reported to be associated with NK cell homeostasis, expansion, and activation (IFN-γ, IL-2, and IL-15) were increased in these analyses. We performed a pathway analysis (Supplemental Figure 4C) on these protein data, and it revealed 7 key pathways differentially increased in participants who developed PGD — notably, the allograft rejection pathway.

**Figure 3:**
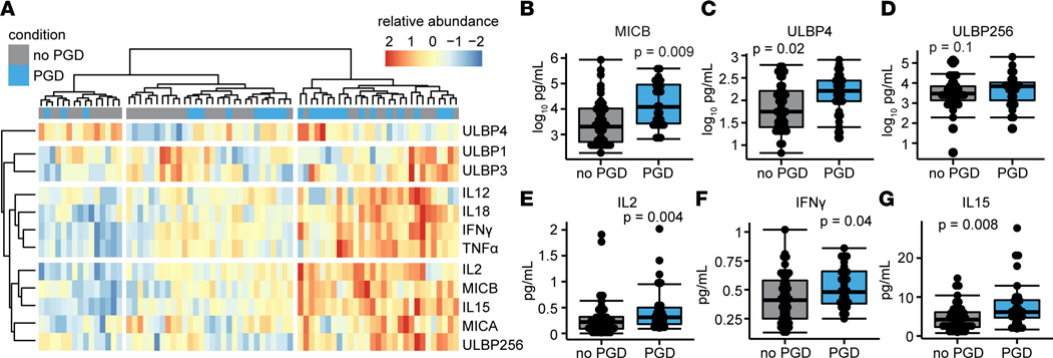
NK cell cytokines and growth factors and NKG2D stress molecule proteins are increased in BAL during severe PGD. BAL from 29 participants with severe PGD and 45 participants without PGD was assayed for 6 NK cell–related cytokines and 6 NKG2D receptor stress molecules on postoperative day 1 after lung transplantation. (**A**) A heatmap demonstrates the fold difference in normalized abundance of these soluble proteins across lung transplant recipients. (**B**–**G**) MICB, ULBP4, ULBP2/5/6, IL-2, IFN-γ, and IL-15 were increased by multiplex ELISA. Summary data are displayed with box-and-whisker plots illustrating individual data points, bounded by boxes at 25th and 75th percentiles, and with medians depicted by bisecting lines. Individual *P* values are shown, and differences were assessed with Mann Whitney *U* test adjusted with the Benjamini-Hochberg procedure.

We hypothesized that NKG2D receptor stress molecules, and cytokines and cell growth factors important to NK cells, would be increased in the BAL of participants who developed severe PGD. Figure 3A shows the heatmap results of our more-focused protein analysis, where we quantified 6 human NKG2D stress ligands (MHC class I polypeptide related protein A [MICA], MICB, UL-binding protein 1 [ULBP1], ULBP2/5/6, ULBP3, ULBP4), in addition to the previously measured cytokines (TNF-α, IFN-γ), and cell growth factors (IL-2, IL-12, IL-15, IL-18). Relative protein abundance is notably increased across all variables in clusters predominantly containing samples from recipients who developed severe PGD. Of the NKG2D stress molecules, MICB was increased in the BAL of participants with severe PGD (Figure 3B; median concentration 59 pg/mL; interquartile range [IQR], 31–142 pg/mL; adjusted *P* = 0.009) compared with participants without PGD (median 27 pg/mL; IQR, 15–53 pg/mL). Participants with PGD also had increased ULBP4 (Figure 3C; median 9.1 pg/mL; IQR, 7.2–11.5 pg/mL; adjusted *P* = 0.02) compared with participants with no PGD (ULBP4; median 5.7 pg/mL; IQR, 4–7 pg/mL) . There was also a trend for increased BAL ULBP2/5/6 among participants with PGD (Figure 3D; median 47 pg/mL; IQR, 23–50 pg/mL; adjusted *P* = 0.1) that did not cross our predefined level of significance. Of the NK cell–related proteins, IL-2 (Figure 3E; median 0.3 pg/mL; IQR, 0.2–0.5 pg/mL; adjusted *P* = 0.004), IFN-γ (Figure 3F; median 0.48 pg/mL; IQR, 0.42–0.52 pg/mL; adjusted *P* = 0.04), and IL-15 (Figure 3G; median 6.2 pg/mL; IQR, 4.7–8; adjusted *P* = 0.008) were increased in BAL during severe PGD relative to BAL of participants without PGD (IL-2, median 0.2 pg/mL; IQR, 0.09–0.32 pg/mL; IFN-γ, 0.41 IQR, 0.2–0.43 pg/mL; IL-15, 4.2 IQR, 2.3 – 6.1 pg/mL).

### Increased BAL NKG2D ligand MICB is associated with ICU morbidity and lung allograft dysfunction.

**Figure 4:**
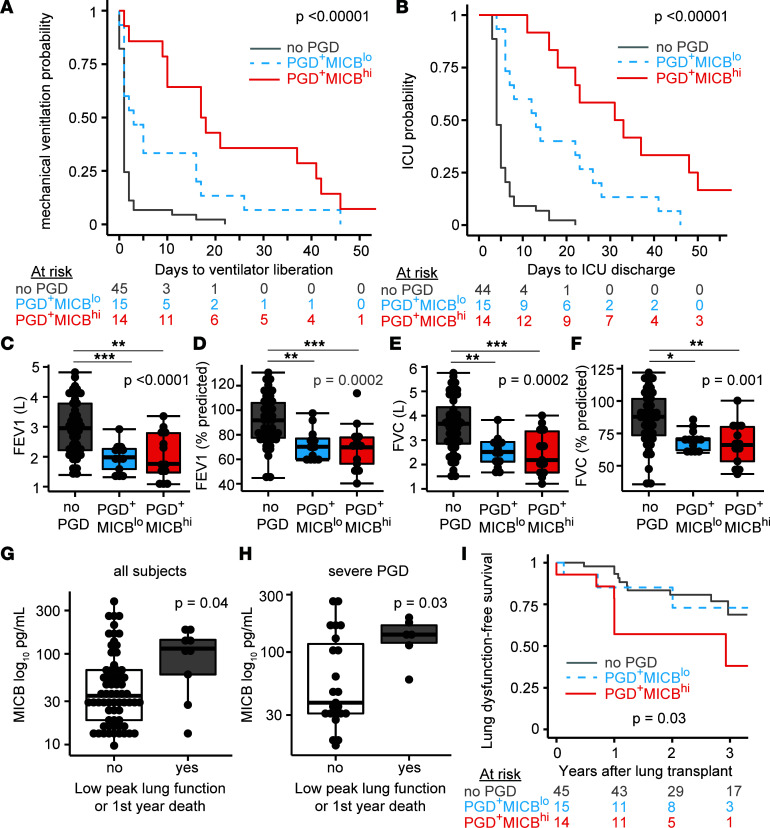
MICB NKG2D stress molecule is associated with mechanical ventilation time and ICU LOS. Participants were stratified by the presence of PGD and greater-than-median MICB (PGD^+^MICB^hi^, *N* = 14), the presence of PGD and lower-than-median MICB (PGD^+^MICB^lo^, *N* = 15) and participants without PGD (*N* = 45). (**A**) Kaplan-Meier plot of mechanical ventilation time among the 3 groups. (**B**) Kaplan-Meier plot of ICU LOS among the 3 groups. (**C**) Forced expiratory volume in 1 second (FEV_1_) in liters. (**D**) FEV_1_ as percent predicted of recipient values. (**E**) Forced vital capacity (FVC) in liters. (**F**) FVC as percent predicted of recipient values. (**G** and **H**) A composite outcome of moderate impairment in peak lung function and death shown for the whole cohort and participants with severe PGD. (**I**) Kaplan-Meier plot of freedom from this composite end point. Summary data are displayed with box-and-whisker plots illustrating individual data points, bound by boxes at 25th and 75th percentiles, and with medians depicted with bisecting lines. Individual *P* values are shown, and differences were assessed by log-rank test for Kaplan-Meier plots (**A**, **B**, and **I**), Kruskal-Wallis test with Dunn test for post hoc analysis (**C**–**F**), and Mann Whitney *U* test (**G** and **H**). *P < 0.05, **P < 0.01, ***P < 0.001.

Given that NK cell and stress molecule genes and proteins were increased in the BAL of lung transplant recipients who developed severe PGD, we hypothesized that the most differentially abundant protein, MICB, would be associated with poor clinical outcomes. We measured the association of MICB with early transplant morbidity via survival models of mechanical ventilation time and ICU LOS. Figure 4A shows Kaplan-Meier plots of mechanical ventilation time among participants with PGD stratified by median BAL MICB concentration compared with participants without PGD. Recipients who developed PGD and had greater than median BAL MICB were 5-fold less likely to be extubated at any time (*N* = 14; PGD^+^MICB^hi^; HR, 5.2; 95% CI, 2.3–11.6; *P* < 0.00001), and a similar risk for need of invasive mechanical ventilation was observed in recipients who developed PGD with lower-than-median BAL MICB (*N* = 15; PGD^+^MICB^lo^; HR, 2.4; 95% CI, 1.5–6.5; *P* = 0.002) compared with recipients who did not develop PGD (*P* = 45). Consequently, lung transplant recipients with PGD and greater-than-median BAL MICB had a nearly 18-fold increased risk of being in the ICU at any time (Figure 4B; HR, 17.9; 95% CI, 6.3–50.6; *P* < 0.00001), and recipients with PGD and lower-than-median BAL MICB had a similar 3-fold risk of need for invasive mechanical ventilation (HR, 3; 95% CI, 1.1–7.9; *P* = 0.03) compared with recipients who did not develop PGD. In an alternate analysis, MICB as a continuous variable remained a risk factor for need of invasive mechanical ventilation (HR, 1.06; 95% CI, 1.02–1.09; adjusted *P* = 0.01) and need for intensive care (HR, 1.05; 95% CI, 1.01–1.11; adjusted *P* = 0.02).

We next asked if early detection of high MICB was associated with reduced peak lung function. We assessed peak lung function within the first year after transplantation, since reduced function has been associated with poor long-term survival (25). Indeed, 2 measures of lung function, forced expiratory volume in 1 second (FEV_1_) and forced vital capacity (FVC), were reduced in participants with PGD and elevated BAL MICB levels within the first year after lung transplantation (Figure 4, C–F). FEV_1_ as a percent of predicted based on recipient values (Figure 4D) was reduced in PGD^+^MICB^hi^ (median 69.5%; IQR, 56.3%–77.8%, *P* = 0.0008) and PGD^+^MICB^lo^ (median 70.2%; IQR, 60.3%–77%, *P* = 0.004) recipients relative to those without PGD (median 91.8%; IQR, 77.5%–106%). Similarly, FVC as a percent of predicted values (Figure 4F) was also reduced in PGD^+^MICB^hi^ (median 66.1%; IQR, 53.4%–80%, *P* = 0.0008) and PGD^+^MICB^lo^ (median 70.5%; IQR, 62%–71.1%, *P* = 0.004) recipients relative to those without PGD (median 87.8%; IQR, 73.5%–101.7%). Notably, three-fourths of the participants with severe PGD had FEV_1_ or FVC lung function values below those considered normal.

We assessed BAL MICB concentrations among individuals with a poor response to lung transplantation, defined as moderate-to-severely reduced peak lung function or first-year mortality. Among all lung transplant recipients, those who did not meet this composite end point had lower BAL MICB (Figure 4G; median 34.4 pg/mL; IQR, 18.6–63.8 pg/mL, *P* = 0.04) relative to the 9 recipients who had a poor transplant response (median 113.9 pg/mL; IQR, 59.2–142.3 pg/mL). Interestingly, this effect was almost exclusively observed in participants with severe PGD (Figure 4H; *P* = 0.03), suggesting that this marker may discriminate long-term outcomes. Figure 4I displays a Kaplan-Meier plot of survival from graft dysfunction, defined as defined as moderately impaired peak lung function, chronic lung allograft dysfunction, or death. Like our early transplant findings, the PGD^+^MICB^hi^ group demonstrated increased risk of graft dysfunction or death (HR, 3.2; 95% CI, 1.2–8.6; adjusted *P* = 0.02).

### Hypoxia induces NKG2D stress ligands on human airway epithelial cells.

**Figure 5:**
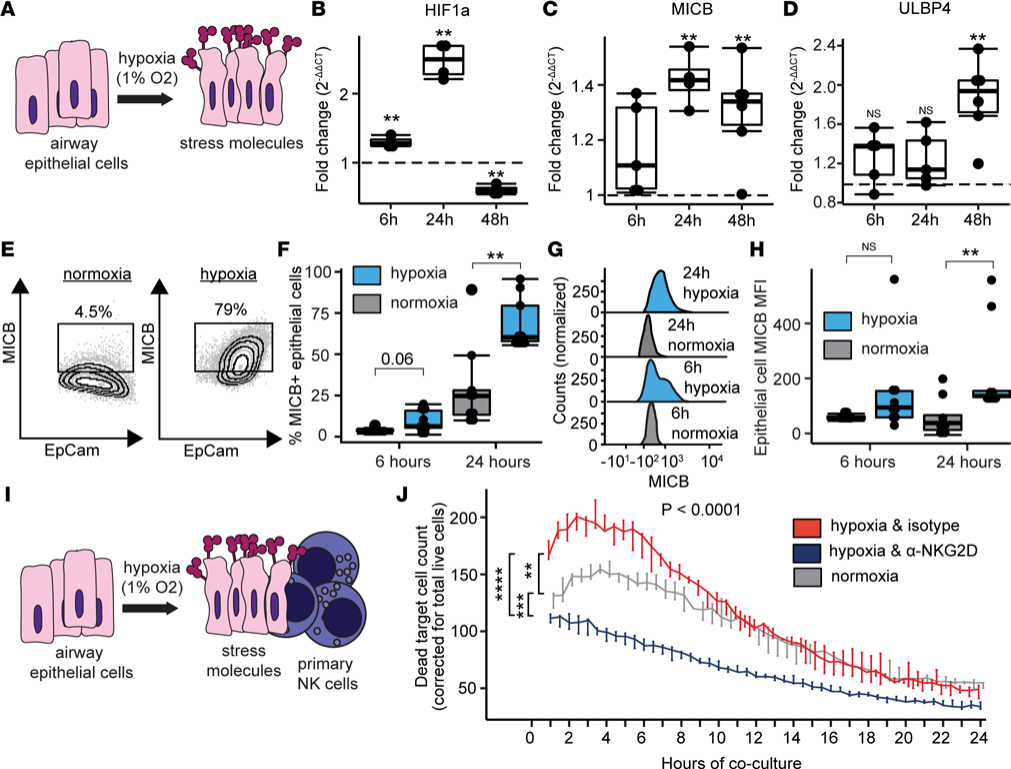
NKG2D stress ligands mediate killing of airway epithelial cells. (A) Airway epithelial cells grown in liquid culture were subjected to hypoxia (1% O_2_) or normoxia (21% O_2_). (**B**–**D**) Transcription at 6 hours (6h), 24 hours (24h), and 48 hours (48h) was measured by PCR for hypoxia inducible factor 1A (HIF1A), MICB, and ULBP4. (**E**) Representative flow cytometry plots of surface MICB with 24 hours of normoxia or hypoxia. (**F**) Airway epithelial cell surface expression of MICB. (**G**) Representative histograms of MICB median fluorescence (MFI). (**H**) MICB MFI is shown at 6 and 24 hours. (**I**) To assess NK cell killing of airway epithelial cells, hypoxic or normoxic control cells were cocultured in a 2:1 ratio for 24 hours with primary human NK cells. Hypoxic cells were treated with isotype-matched control antibody or anti-NKG2D blocking antibody for 24 hours preceding the experiment. (**J**) Dead cell counts are shown at 30-minute intervals across 24 hours comparing total AUC for the 3 conditions. Summary data are displayed with box-and-whisker plots illustrating individual data points, bounded by boxes at 25th and 75th percentiles, and with medians depicted with bisecting lines. Individual *P* values calculated with Mann Whitney *U* test (**B**, **C**, **D**, **F**, and **H**) and 1-way ANOVA with Tukey’s honestly significant difference post hoc comparisons. ** *P* < 0.01, *** *P* < 0.001, **** *P* < 0.0001.

We further hypothesized that stress molecules were originating from injured donor lung epithelial cells. To investigate this hypothesis, we employed an in vitro system to model PGD (Figure 5A) (26). Reports have suggested that NKG2D stress ligands are downstream of hypoxia inducible factor 1-α signaling (*HIF1A*) (27). We measured *HIF1A*, *MICB*, and *ULBP4* mRNA in airway epithelial cells at 6 hours, 24 hours, and 48 hours of hypoxia (1% O_2_). We found maximum induction of *HIF1A* at 24 hours (Figure 5B; *P* = 0.001). Similarly, *MICB* was maximally induced at 24 hours of hypoxia (Figure 5C; *P* = 0.003) but remained elevated at 48 hours (*P* = 0.008). *ULBP4* mRNA was increased at 48 hours of hypoxia (Figure 5D, *P* = 0.02). Supplemental Figure 5 depicts measurements of *HIF1A* downstream targets in participant BAL, as well as the in vitro measurements of 6 additional human NKG2D stress molecules, with induction in at least 1 time point within 48 hours for all molecules except *ULBP3*. Participants with severe PGD had a trend for increased HIF1A pathway gene scores (*P* = 0.25), with this difference largely driven by *HIF1A* and *VEGFA* transcription.

We additionally hypothesized that MICB and other stress molecules would be increased on the cell surface of airway epithelial cells in response to the stress of hypoxia. The flow cytometry gating strategy is depicted in Supplemental Figure 6 with individual MICB contour plots shown in Figure 5E. In parallel to the transcriptional findings, we found increased MICB on epithelial cell surfaces after 24 hours of hypoxia relative to normoxic culture conditions (Figure 5F; *P* = 0.002). Representative histograms (Figure 5G) illustrate the observation of increased epithelial cell surface density by MICB median fluorescence intensity (MFI) at 24 hours of hypoxia (Figure 5H; *P* = 0.01). Additional NKG2D stress ligands were measured by flow cytometry on hypoxic airway cells, where we found increased surface expression of ULBP1 and ULBP2/5/6 (Supplemental Figure 6, B–E).

### NK cell killing of hypoxic airway epithelial cells is mediated by the NKG2D receptor.

We assessed if primary airway epithelial cells exposed to hypoxia would have increased susceptibility to NK cell killing via the NKG2D receptor. Following 24 hours of hypoxia or normoxia, primary airway epithelial cells from healthy donors were cocultured in normoxic conditions for an additional 24 hours with primary NK cells (Figure 5I). The flow cytometry gating strategy is illustrated in Supplemental Figure 7A with representative contour plots for the 3 conditions in Supplemental Figure 7B. We found increased killing of hypoxic airway epithelial cells by NK cells across all concentrations (Supplemental Figure 7C). Most notably, in 2:1 NK cell/epithelial cell cocultures, increased killing was observed among cocultures with hypoxic epithelial cells, as compared with cocultures with normoxic epithelial cells (*P* = 0.02) or cocultures with hypoxic airway cells, but pretreatment with NKG2D blocking monoclonal antibody (*P* = 0.03). Alternatively, total cytotoxicity, as measured by area under the killing curve, was increased in NK cells cocultured with hypoxic NK cells relative to the other 2 conditions (Supplemental Figure 7D). Using live cell culture imaging, we examined primary airway cell killing over 24 hours when cultured at a ratio of 2:1 NK cells/epithelial cells (Figure 5J). Notably, pretreatment with NKG2D blocking antibody blunted the killing at all time points as compared with isotype-matched control Ig-treated NK cells (*P* = 0.00003) and wells with normoxic cells (*P* = 0.0002). Since T cells also express NKG2D, we also assessed if they killed hypoxic airway epithelial cells in an NKG2D-dependent fashion. We observed no differences in airway cell cytotoxicity under several concentrations of T cells pretreated with NKG2D blockade or isotype control (Supplemental Figure 8). These findings suggest that NK cell killing of stressed airway cells is mediated through the NKG2D receptor.

### BAL NETs are associated with early injury and correlate with NK cell pathway cytokines.

**Figure 6:**
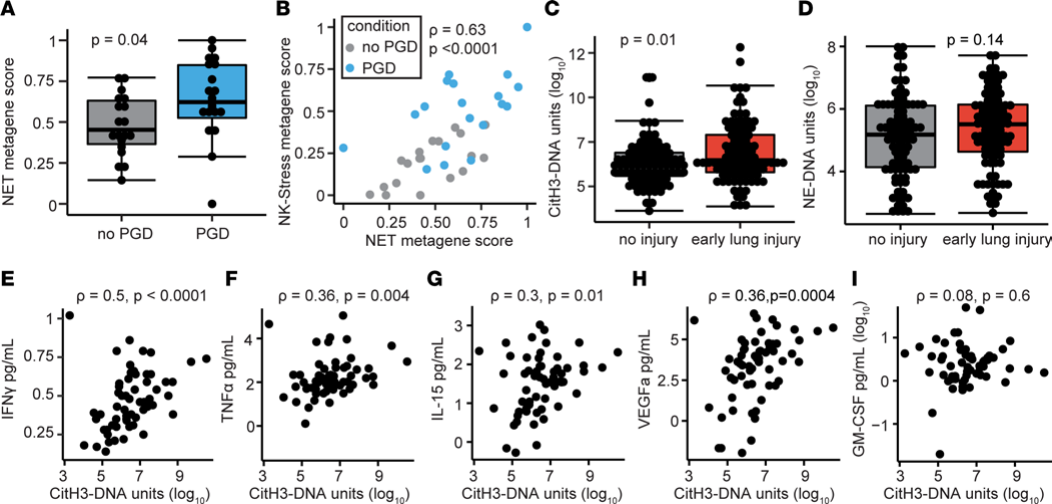
Neutrophil extracellular traps are increased early after transplant and correlate with NK cell cytokines. (**A**) NET metagene score is increased in severe PGD. (**B**) NET metagene score correlates with the NK-stress metagene score. (**C**) CitH3-DNA complexes are increased in grade 2 or 3 PGD at POD1. (**D**) NE-DNA complexes are shown at POD1 in grade 2 or 3 PGD compared with grade 0 or 1 PGD. (**E**–**I**) CitH3-DNA complexes are correlated with NK cell cytokines IFN-γ, TNF-α, and IL-15, but not with GM-CSF or VEGF-A. Summary data are displayed with box-and-whisker plots illustrating individual data points, bound by boxes at 25th and 75th percentiles, and with medians depicted with bisecting lines. Individual *P* values are shown, and differences were assessed with Mann Whitney *U* test and correlations with Spearman’s test.

We hypothesized that increased NET transcripts would be present in the BAL of lung transplant recipients with severe PGD. Supplemental Table 5 shows the demographic information for this cohort. Indeed, a NET metagene score was increased in participants with severe PGD compared with those without PGD (Figure 6A; *P* = 0.04). Interestingly, there was significant correlation between the NK-stress gene score and the NET gene score (Figure 6B; Spearman’s ρ = 0.63, *P* < 0.0001). Recipients with severe PGD tended to have increased NET and NK cell gene signatures. We next measured NETs using 2 custom ELISAs (neutrophil-elastase–DNA [NE-DNA] and citrullinated histone 3–DNA [citH3-DNA] complexes). We found that citH3-DNA complexes were associated with early allograft injury, defined as severe PGD on POD1 (Figure 6C; *P* = 0.01). NE-DNA complexes were also increased in recipients with severe PGD on POD1 but did not cross our prespecified level of significance (Figure 6D; *P* = 0.14). We hypothesized that NK cells may release cytokines that influence the production of NETs. In the BAL, we examined the association between citH3-DNA complexes and cytokines that either stimulate NK cells (IL-15) or can be produced by NK cells (IFN-γ, TNF-α, GM-CSF, VEGF-A). We found that IFN-γ had the strongest association with NETs (Figure 6E; Spearman’s ρ = 0.5, *P* < 0.0001), but TNF-α (Figure 6F; ρ = 0.36), IL-15 (Figure 6G; ρ = 0.3), and VEGF-A (Figure 6H; ρ = 0.36) — not GM-CSF (Figure 6I, ρ = 0.08) — were positively correlated with NETs.

### Cytokines secreted by activated NK and effector cells induce NETs in a model system.

**Figure 7:**
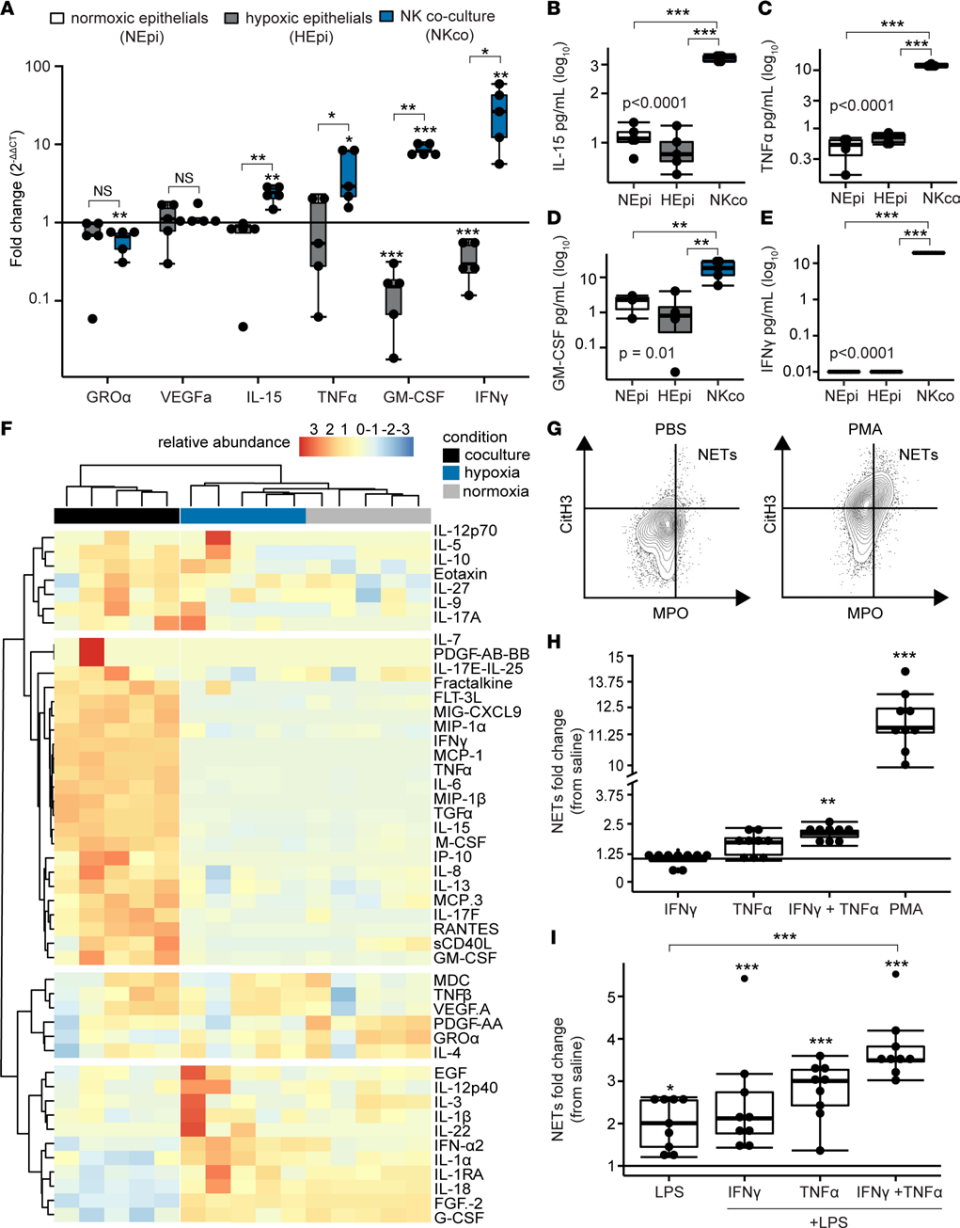
NK cell cytokines induce NETs. Airway epithelial cells were cultured for 24 hours under normoxic (NEpi, *N* = 5) or hypoxic (HEpi, *P* = 5) conditions, and cocultured with NK cells after hypoxia (NKco, *N* = 5). (**A**) Cytokine transcripts were measured from each condition. (**B**–**E**) Soluble concentrations of cytokines were measured for IL-15, TNF-α, GM-CSF, and IFN-γ. (**F**) A heatmap displays the relative abundance of cell culture proteins measured within a 70-analyte panel across the 3 conditions. Neutrophil extracellular traps (NETs) were measured with flow cytometry (*N* = 9 per condition). (**G**) Representative dot plots are displayed for negative (saline) and positive (PMA) controls. (**H**) NET fold-change relative to saline controls are shown for neutrophils stimulated with NK cell cytokines. (**I**) NET fold-change were assessed with neutrophils primed with NK cell cytokines and stimulated with LPS. Summary data are displayed with box-and-whisker plots illustrating individual data points, bound by boxes at 25th and 75th percentiles, and with medians depicted with bisecting lines. Individual *P* values are shown, and differences were assessed with Mann Whitney *U* test after Benjamini-Hochberg correction for multiple comparisons. *P < 0.05, **P < 0.01, ***P < 0.001. NEpi, normoxic epithelial cells; HEpi, hypoxic epithelial cells; NKco, NK cell coculture.

We returned to our coculture in vitro model system and measured mRNA of target cytokines for normoxic epithelial cells, hypoxic epithelial cells, and from coculture of NK cells with hypoxic epithelial cells. Figure 7A shows that transcripts of IL-15 (*IL15*), TNF-α (*TNFA*), GM-CSF (*GMCSF*), and IFN-γ (*IFNG*) were all increased in the NK cell coculture group relative to normoxic epithelial cells and hypoxic epithelial cells. Notably, in coculture conditions, IFN-γ demonstrated 26-fold (IQR, 12.3–42.6) induction. We did not find increased transcription of GRO-α (*CXCL1*) or VEGF-A (*VEGFA*). We further hypothesized that we would detect soluble proteins in a similar pattern across these conditions. We focused on the proteins with increased gene transcription and found that IL-15 (Figure 7B), TNF-α (Figure 7C), GM-CSF (Figure 7D), and IFN-γ (Figure 7E) were all increased relative to hypoxic airway epithelial cells and normoxic airway epithelial cells. Again, IFN-γ and TNF-α showed the highest concentrations in the NK cell coculture conditions. In a multiplex protein analysis of cell culture supernatant, there were multiple differentially abundant cytokines and chemokines in NK cell cocultures relative to hypoxic airway epithelial cells or normoxic airway epithelial cells (Figure 7F).

To assess if cytokines produced by NK cells induce neutrophils to release NETs, as assessed by CitH3, we cultured primary human neutrophils with IFN-γ and TNF-α. Supplemental Figure 9A shows the gating strategy for this flow cytometry experiment, and representative dot plots are displayed in Figure 7G. Indeed, we found that the combination of IFN-γ and TNF-α resulted in a 2.1-fold (IQR, 1.9–2.2) induction of NETs relative to the saline control (Figure 7H; *P* = 0.003). Relative induction (Figure 7I) and NETs as a percentage of neutrophils (Supplemental Figure 9B) were lower than stimulation achieved with PMA. Therefore, we hypothesized that NK cell cytokines may prime neutrophils to release NETs. We cultured primary human neutrophils with cytokines and then stimulated with LPS. Compared with saline alone, priming with IFN-γ, TNF-α, or both cytokines followed by LPS stimulation significantly induced NETs relative to the negative control (Figure 7I). The priming of neutrophils with both IFN-γ and TNF-α yielded an increase in NETs relative to LPS alone.

## Discussion

Our results across a large multicenter lung transplant cohort demonstrate the importance of NK and T cell pathways and NKG2D receptor stress molecules in the development of severe PGD. Furthermore, we demonstrate that increased soluble stress molecules are associated with prolonged time to extubation, increased ICU LOS, and adverse long-term clinical outcomes. In vitro experiments show that NK cell recognition of NKG2D ligands is mechanistically linked to stressed airway epithelial cell killing, a key pathophysiologic finding in PGD. Finally, we provide evidence that NK cells and neutrophils are correlated and may interact to initiate an innate immune response in the lungs, driving lung injury after transplantation. These findings reveal potentially novel pathways that may be targeted by clinical intervention to prevent or treat PGD.

Up to one-third of all lung transplant recipients develop severe PGD, which, as we have shown here, increases risk for early mortality. Interestingly, severe PGD grades during the first 48 hours after transplantation are not always associated with mortality. Rather, there is evidence of distinct clinical latent classes, whereby survival is worse among participants with nonresolving early inflammation or those with late severe PGD (28). Here, we describe MICB as a key early marker of lung allograft injury that distinguishes risk for severe PGD. Furthermore, we found that increased BAL NKG2D stress molecules collected on POD1 characterized risk for long-term clinical outcomes among participants who developed severe PGD. These data suggest that measuring BAL MICB early after transplantation could be effective in identifying patients at high risk for PGD, allowing for targeted therapies.

Stress molecules are increased in response to a variety of injuries and are ligands for the NKG2D receptor, found on NK cells and T cells (29). Interestingly, there is significant heterogeneity in NKG2D stress ligand tissue expression (30). Here, we describe increases in MICB and ULBP4 in posttransplant BAL, but we show robust increases of ULBP2 and ULBP5 on hypoxic airway cells. Investigation into airway epithelial cell type propensity for NKG2D ligand expression may reveal additional insights into this disease pathogenesis. Notably, RNA-Seq among human samples showed relatively low transcription of MICB. We posit that this is due to the rapid and diverse regulation of this gene (30). We noted decreased transcription at 48 hours of MICB in our in vitro studies with hypoxia. By the time of bronchoscopy, > 48 hours may have transpired since the donor organs initially experienced ischemic insult. We also found temporal dissociation between transcription of some of the NKG2D stress ligands and HIF1A, suggesting additional mechanisms for induction, such as through TLR signaling (31, 32). MICA and MICB are highly polymorphic, and donor mismatches may generate recipient adaptive immune responses. Antibodies against MICA have been associated with chronic lung allograft dysfunction (CLAD) (33). Whether these antibodies are generated during early acute inflammation, such as PGD; are markers of ongoing lung injury; or are pathogenic remains to be elucidated. Thus, the study of donor and recipient NKG2D receptor and ligand binding may reveal additional mechanisms of allograft injury.

In our clinical analyses, we found a trend for association between the pretransplant diagnosis of ILD and PGD. Notably, our multicenter cohort performs more transplants for ILD than the national average, and recipients with ILD may experience more secondary pulmonary hypertension, a risk factor for PGD. Additionally, ILD may result in increased NK cell activation. Several groups have shown that increased BAL NK cells are associated with a worse ILD prognosis (34). In experimental models of small airway fibrosis, NK cells are increased at the sites of injury, and their depletion may blunt the fibrotic process (35, 36). Furthermore, it is well established that NK cells, especially in response to cytomegalovirus infection, may have adaptive or memory-like traits (37). In a similar fashion, chronic NKG2D activation may influence the education of NK cells and alter their response to a variety of stimuli (38). As such, it is possible that the NK cells in participants with fibrotic lung diseases may be primed for activation and subsequent PGD. Alternatively, the stressed epithelium in retained fibrotic upper airways may be more susceptible to hypoxic injury and release of NKG2D stress ligands such as MICB.

We have previously shown in experimental models of PGD that NKG2D ligands are increased on pulmonary endothelial and epithelial cells and that genetic deletion or blockade of this receptor on NK cells reduces lung ischemia-reperfusion injury (15). Here, we support these findings using human samples (discovering the major stress ligands for NKG2D) and an in vitro model of airway cell hypoxia. We showed that blockade of the NKG2D receptor on NK cells prevented stressed airway cell death in our model coculture system. Notably, we also identified that CD8^+^ and CD4^+^ T cell gene transcripts were increased during severe PGD. There is an overlap between T cell and NK cell gene signatures, and it is likely that injury results from the contributions of each of these cells (39). Moreover, CD8^+^ and γδ T cells also express NKG2D that can recognize cognate ligand. However, as opposed to NK cells, where NKG2D is a potent activating signal, NKG2D activation of T cells does not occur in isolation and at least requires a second signal through the T cell receptor (40). Moreover, it has been reported that NKG2D engagement may not be sufficient for CD8^+^ conventional T cell costimulation and may only occur under restricted circumstances or through modulation of CD28 (41). Our in vitro experiments with NKG2D blockade of T cells further support this concept. Nonetheless, further investigation could discern the dominant cell types in this pathway during PGD and any contributions from an NKG2D^+^ T cell component. However, the clinical translation of these findings through the treatment of transplant recipients with an existing anti-NKG2D monoclonal antibody as part of an induction regimen would broadly target this pathway regardless of cell type (42).

Our data build on prior work showing that PGD pathophysiology is marked by a biphasic injury response. Early studies of donor lung tissue and BAL shortly after reperfusion showed altered innate lymphoid cell distributions and increased TLR gene transcription (6, 43). Experimental animal models of PGD have identified key roles for neutrophils and NK cells in PGD pathophysiology (15, 16, 44). Neutrophils are activated via recognition of mitochondrial DNA by TLR9 and cause lung injury via the release of NETs that destabilize the lung barrier (17, 45, 46). Here, we show that NETs are associated with early high-grade PGD, correlated to BAL cytokines associated with NK and other cells, and they could be induced by IFN-γ and TNF-α. This suggests a linkage between NK cell activation and NETosis and indicates that NK cells may bridge the early and late phases of PGD inflammation. Other groups have demonstrated that IFN-γ and NK cells induce NETosis during venous thrombosis; however, further study in animal models of transplant are needed to establish the mechanistic underpinnings of our provisional findings in human samples (20, 47). Moreover, there may be additional interactions not categorized here, such as through contact-dependent means or trogocytosis (48, 49).

Our study is not without limitations. While samples were collected in a multicenter cohort, other centers may have differing demographics and clinical protocols. As such, validation of these data across broader cohorts would bolster these conclusions. Samples were not available from many patients for a variety of reasons, including being too hypoxemic to undergo a BAL, which may have skewed our cohort to a less severe phenotype. Biosample analyses were performed in nested subsets, and matching between cases and controls was performed; however, it serves that additional insight may be gained from analyses within the full cohort. We were limited to a single sample-collection time point, as participants without PGD are extubated quickly and those with severe PGD are at increased risk from additional research bronchoscopies. Cell culture hypoxia may not be fully representative of ischemia-reperfusion injury, since tissue oxygenation may be higher than our experimental conditions (50). However, we found multiple similarities between BAL fluid composition and hypoxia cell culture supernatant, suggesting the presence of parallel processes. We focused on innate immune pathways, but our results also indicate strong signals for adaptive immune cell involvement even in the very early clinical course posttransplantation that will be investigated in future work. Plasma was also collected at the time of BAL, but we focused on BAL samples for this study, as previous work by our group has shown a stronger signal in this compartment (16).

This study also has notable strengths. Samples were prospectively collected from the target organ across a large multiple center cohort at a pathophysiologically critical time. Our observational findings had biologic validation with in vitro models. Finally, our findings in human disease are strengthened by parallel results in preclinical animal models.

In conclusion, we describe a role for NK cell pathways and NKG2D receptor ligands in human PGD. These findings improve our understanding of PGD pathogenesis and set the stage for early phenotyping of patients and targeted therapeutics to improve short- and long-term clinical outcomes after lung transplantation.

## Methods

### Study design.

All consenting adult lung transplant recipients who underwent single-, double-, or heart-lung transplantation at UCSF and UCLA from January 2, 2016, to February 20, 2020, were enrolled. BAL was collected on POD1 during routine bronchoscopy using 1–2 aliquots of 20 mL saline, typically in the lingula or right middle lobe. Blood was also collected at the time of BAL sampling. Supplemental Figure 1 depicts the inclusion and exclusion criteria for the analyses performed.

### Clinical data, outcomes, protocols.

Data abstracted from the medical charts include information on demographics, transplant indication, hospital course, spirometry, and survival. PGD was graded on POD0–POD3 according to international criteria (21). Severe PGD was defined as grade 3 disease on POD2 or POD3 (22). Participants on extracorporeal membrane oxygenation support after transplant were assigned grade 3. The term “no PGD” was applied to recipients with PGD grade 0 or 1 on POD2 and POD3. We labeled severe PGD grades on POD1 as “early allograft injury.” Freedom from mechanical ventilation was defined as days from transplant until participants were extubated. All lung transplant recipients were admitted to the ICU postoperatively. ICU LOS was defined as the number of days between the date of transplant surgery and the date of transfer to a lower-acuity bed. Peak lung function was defined as the average of the best 2 values of FEV_1_ or FVC within the first year after transplantation (51). Low-peak lung function was considered < 80% FEV_1_ and < 80% FVC, calculated using recipient characteristics, and moderate lung function impairment was defined as FVC or FEV_1_ < 60% (52, 53). CLAD was defined according to established criteria as an unresolving 20% decline in FEV_1_ or FVC lasting over 30 days (54) and was determined from clinical records, as previously described (55, 56). To assess lung dysfunction in this cohort with shorter follow-up and poor peak lung function, we generated a combined end point of time to moderate lung function impairment, CLAD, or death.

Standard induction regimens for all participants included methylprednisolone, mycophenolate mofetil, and basiliximab; antithymocyte globulin was used for combined heart and lung transplant recipients in lieu of basiliximab. Initial maintenance immunosuppressant therapy included tacrolimus, prednisone, and mycophenolate mofetil.

### RNA-Seq.

After BAL collection, RNA was preserved in RNAlater at –80°C (Thermo Fisher Scientific, AM7020). Total RNA was extracted with the miRNeasy Kit (Qiagen), and quality was confirmed via NanoDrop (Thermo Fisher Scientific) and Agilent 5200 Fragment Bioanalyzer (Agilent Technologies). Total RNA was sent to the UCSF Genomics CoLab for library preparation and sequencing. Samples were enriched with the NEBNext Isolation Module (New England Biolabs). Illumina compatible RNA-Seq libraries were generated from RNA using the Tecan Universal Plus mRNA-Seq kit. Paired-end Illumina sequencing of 100 nucleotide base pairs was performed on an Illumina NovaSeq 6000. Alignment to the Ensembl gene build 95 of the human reference genome was performed in STAR. After alignment, gene counts were normalized in DESeq. Outliers were excluded based on principal component analysis using Tukey’s fence criteria (k > 3). Metagene values were calculated using the singscore R module and with validated cell gene lists (Supplemental Table 6) (57).

### BAL proteomics and NETs assays.

Soluble NKG2D stress molecules were quantified with a custom 6-plex Luminex-based assay (R&D Systems, Eve Technologies). BAL cytokine and chemokine proteins were similarly measured with a 71-analyte multiplex assay (Discovery Assay, Eve Technologies), and cell culture proteins were measured with a 48-analyte multiplex assay (Discovery Assay, Eve Technologies). NETs in BAL and cell culture were quantified by ELISA, as previously reported (16, 19).

### Primary NK cell and T cell isolation and culture.

Primary human NK cells were obtained from AS and sourced from blood from healthy, consenting donors or from plateletpheresis leukoreduction filters (Vitalant) as described (58). NK cells were isolated from primary sources using a negative selection kit RosetteSep Human NK Cell Enrichment Cocktail (catalog 15065, STEMCELL Technologies) according to the manufacturer’s protocol. The resulting NK cells were grown in culture media containing GMP SCGM (CellGenix) supplemented with 1% L-glutamine, 1% penicillin and streptomycin, 1% sodium pyruvate, 1% nonessential amino acids, 10 mM HEPES, and 10% human serum (heat-inactivated, sterile-filtered, male AB plasma; all from MilliporeSigma). NK cell purity was confirmed with flow cytometry as CD3^–^CD56^+^CD16^+^, as previously shown (58). T cells were obtained from healthy consenting donors and grown as previously described (59).

### Primary airway epithelial cell isolation and culture.

For reverse transcription PCR, NK cell coculture, and NK cell cytotoxicity experiments, primary airway epithelial cells were obtained and cell culture techniques were applied as described previously (60). Human tracheal sections were obtained from excess explanted donor lung tissue. Airways were washed in PBS with antibiotics, and mucous was digested with 5 mM dithiothreitol. Tissue was digested with 0.1% protease in PBS at 4°C overnight, followed by suspension in 5% FBS in DMEM. Cells were centrifuged for 6 minutes at 300*g* at ambient temperature and resuspended in 0.05% trypsin-EDTA. Trypsin was neutralized with 5% FBS in DMEM, and cells were strained through a 100 μm filter and centrifuged again (centrifuged for 6 minutes at 300*g* at ambient temperature). Cells were grown in submerged Air Liquid Interface media in T25 flasks at 37°C, 5% CO_2_ incubator until 80%–90% confluence for experiments with media changes every 72 hours. For individual experiments, cells were treated with 0.05% trypsin-EDTA and proportioned in wells containing 5% FBS in DMEM.

### Quantitative PCR.

Primary airway epithelial cells were proportioned at 10,00 cells/well in sterile 48-well plates until grown to 80%–90% confluence. Cells were subjected to 6, 24, and 48 hours of hypoxia (1% O_2_) or normoxia (21% O_2_). Total RNA was extracted from cells using QIAzol (Qiagen) and the miRNeasy kit (Qiagen, 217084). cDNA was generated using the SuperScript III First-Strand Synthesis Mix (Invitrogen, 11752250), and Taqman qPCR (Invitrogen) was performed to quantify *HIF1A*, *MICA*, *MICB*, *ULBP1*, *ULBP2*, *ULBP3*, ULBP4 (*RAET1E*), ULBP5 (*RAET1G*), and ULBP6 (*RAET1L*) gene expression.

For coculture experiments, primary human airway epithelial cells were seeded at 10,00 cells/well in sterile round-bottom 96-well plates and allowed to grow to 80%–90% confluence (61). Cells were exposed to hypoxia or normoxia for 24 hours. A group of hypoxic epithelial cells were further cultured in a ratio of 1:2 with primary human NK cells for an additional 24 hours (58). Total RNA was collected, and gene expression was quantified for IFN-γ (*IFNG*), TNF-α (*TNFA*), GRO-α (*CXCL1*), IL-15 (*IL15*), VEGF-A (*VEGFA*), and GM-CSF (*CSF2*). Specific gene expression was compared with housekeeping gene PPIA expression to determine ΔCt or ΔΔCt, as previously described (62).

### Flow cytometry analysis.

Given high cell needs for these experiments, we used the human bronchial epithelial cell line 16HBE14o- (Sigma-Aldrich, SCC150) grown in sterile tissue-culture treated plates. At 80%–90% confluence, cells were exposed for 6 or 24 hours to either hypoxic (1% O_2_) or normoxic (21% O_2_) conditions. Cells were dissociated using 0.25% trypsin (MilliporeSigma, SM-2003-C). Trypsin was neutralized with FBS before the 16HBE cells were collected. Cells were first incubated with Human TruStain FcX (BioLegend, 422302) to block nonspecific binding and with Ghost Dye Violet 510 as a dead cell marker. Epithelial cells were identified as the Brilliant Violet 786–conjugated (BV786-conjugated) anti-CD326^+^ (clone EBA-1, BD Biosciences, 565686) and R-phycoerythrin-cyanine–conjugated (PE-Cy7–conjugated) anti-CD45^–^ (clone HI30, BD Biosciences, 557748) population. Subsets of epithelial cells expressing NKG2D stress molecules were identified using PE-conjugated MICA (clone 159227, R&D Systems, FAB1300P), allophycocyanin-conjugated (APC-conjugated) MICB (clone 236511, R&D Systems, FAB1599A), Alexa Fluor 700–conjugated ULBP1 (clone MM0592-10K10, Novus Biologicals, NBP2-11986AF700), BV421-conjugated ULBP2/5/6 (clone 165903, BD Biosciences, 748128), and FITC-conjugated ULBP3 (polyclonal, Biorbyt, BIRBORB8450). Data were acquired using the FACSAria Fusion Flow Cytometer (BD Biosciences) and analyzed using FCS express version 7 (De Novo Software). NKG2D stress molecules were quantified as a percentage of total epithelial cells and by MFI.

Primary human neutrophils were collected from whole blood from healthy donors and isolated as previously described (63, 64). To assess NK cell cytokine impact on NETosis, neutrophils were cultured for 4 hours with PMA (positive control, 100 nM, Sigma-Aldrich, P1585) or PBS (negative control), TNF-α (100 ng/mL, R&D Systems, 210TA005CF), IFN-γ (100 ng/mL, BioLegend, 570202-BL), or both cytokines. To assess priming for NETs, cytokine-treated or saline-treated neutrophils were stimulated with LPS (5 μg/mL, Sigma-Aldrich, L2880) for 4 hours. To quantify NETs via flow cytometry, cells were stained with DAPI (Invitrogen, D1306), FITC-conjugated anti-myeloperoxidase (anti-MPO) (clone 2D4, Abcam, ab90812), and unconjugated anti-CitH3 (Polyclonal, Abcam, ab5103), followed by secondary anti–rabbit AF594–conjugated (Invitrogen, A11012). Data were collected using the BD LSRII Cytometer and analyzed using FlowJo version 10 (BD Biosciences). Percentage of NETs of live neutrophils were gated according to the strategy outlined in Supplemental Figure 7A.

### Primary NK cell cytotoxicity assay.

Primary human airway epithelial cells were stained with CellTrace Violet (Thermo Fisher Scientific, C34571) and proportioned into 96-well plates at 10,000 cells/well and rested overnight. Cells were exposed to hypoxic (1% O_2_) or normoxic (21% O_2_) culture conditions for 24 hours and then cocultured with primary human NK cells for 24 hours at increasing effector/target ratios. In a treatment trial, NK cells were incubated with 10 μg of blocking anti–human NKG2D monoclonal antibody (clone 1D11, BioXCell, BE0351) or 10 μg of anti–human isotype control antibody (clone MOPC-21, BioXCell, BE0083) before coculture. Cells were collected and stained with Fixable Viability Dye eFluor 660 (Invitrogen, 65-0864-14). Data were collected on the FACSAria Fusion Flow Cytometer. NK cell cytotoxicity was measured by the difference in percentage of live/dead CellTrace Violet^+^ cells between samples. Negative thresholds were set based on unstained and viability controls. Dead epithelial cells were identified as the CellTrace Violet^+^ and eFluor 660^+^ population (Supplemental Figure 6). To visualize live cell killing, primary airway epithelial cells were stained with CytoLight Green (catalog 4705, Sartorius) and plated at a concentration of 5,000 cells per well in a flat-bottom plate. Ten thousand primary human NK cells or T cells were added, along with Cytotox Red reagent (4633, Sartorius). Cell death was recorded and automatically scored with the Incucyte (S3) software in 30-minute intervals across 24 hours.

### Data and materials availability.

Genomics data are available in the European Molecular Biology Laboratory Bioinformatic Institute’s ArrayExpress database (https://www.ebi.ac.uk) under accession no. E-MTAB-12282. All other data are available in the main text, the supplemental materials, or upon request to the corresponding author.

### Statistics.

Unless otherwise specified, data are reported as medians with IQRs. Normality was assessed with the Shapiro-Wilk test. Comparisons between multiple groups were made using Kruskal-Wallis 1-way test with Dunn’s post hoc testing for nonparametric data or 1-way ANOVA with Tukey’s honestly significant test for parametric data. Individual group comparisons were made with the Mann Whitney *U* test. In analyses of multiple comparisons, results were adjusted with the Benjamini-Hochberg procedure. *P* values less than 0.05 were considered significant.

Differences among cohort subject characteristics were compared using 2-tailed Student’s *t* and χ^2^ tests for continuous and categorical variables, respectively. We utilized logistic regression to test the association between our primary predictors of interest and PGD. Primary predictors included donor age (per decade), donor sex, donor smoking > 20 pack years, recipient age at transplant (per decade), recipient sex, ischemic time, transplant type (bilateral versus other), cardiopulmonary bypass as a categorical variable, BMI, transplant indication, pulmonary artery systolic pressure (per 10 mmHg), and lung allocation score (LAS) at the time of transplant. These were adopted from findings in the multicenter Lung Transplant Outcomes Group (2) and are consistent with contemporary recommendations for the selection of covariates based on plausible biologic rationale (2, 65). We completed the < 1.6% of missing data fields with 10-fold multiple imputation. Logistic regression with the same covariates was also used to test the association between PGD and mortality at 90 days or 1 year after transplantation.

We utilized Cox proportional hazards models to test the association between our primary predictors of interest and duration of mechanical ventilation, time to discharge from the ICU, and the combined end point of lung dysfunction–free survival. Proportional hazards were assessed visually and with the Schoenfeld test and martingale residuals. Time to these events were visualized with Kaplan-Meier methods and compared by the log-rank test.

Statistical analyses were performed in R (version 4.1.1, R Foundation for Statistical Computing) using the survminer, plyr, ggplot, survival, stringr, multcomp, and ggpubr packages, and PGD clinical modeling was performed in SAS (version 9.4 SAS institute).

### Study approval.

Written informed consent was received prior to participation in this study by all participants. The UCSF and UCLA IRBs approved this study under protocols 13–10738 and 13-00462, respectively.

## Author contributions

Conceptualization was contributed by MRL and DRC. Methodology was contributed by DRC, JRG, MRL, and LLL. Investigation was contributed by DRC, TT, MM, JJT, EAA, KW, SRH, JAG, LLL, MEK, LEL, NAK, RS, AV, JK, CV, SAC, CRL, BM, EBP, SSW, JK, AA, DMS, JAB, CSC, ASh and ASa. Formal analysis was contributed by DRC, YG, and JPS. Visualization was contributed by DRC. Funding acquisition was contributed by MRL and DRC. Writing of the original draft was contributed by DRC, MRL, and TT. Review and editing of the manuscript were contributed by DRC, MRL, JRG, JPS, JB, SSW, and LLL. Order of authorship was determined based on relative contribution to the overall project.

## Supplementary Material

Supplemental data

## Figures and Tables

**Table 1:**
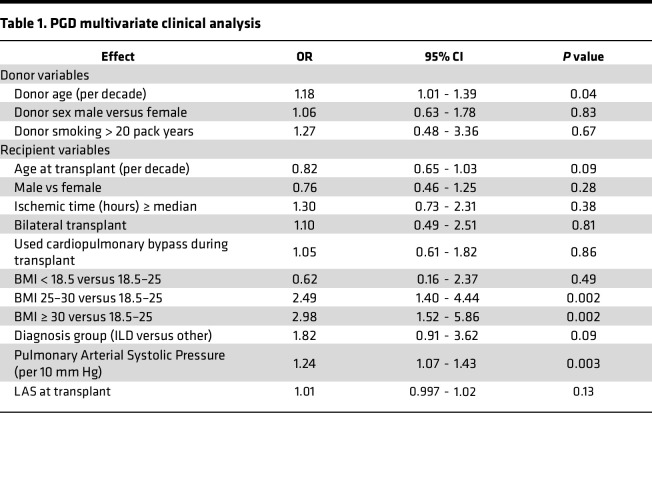
PGD multivariate clinical analysis

**Table 2:**
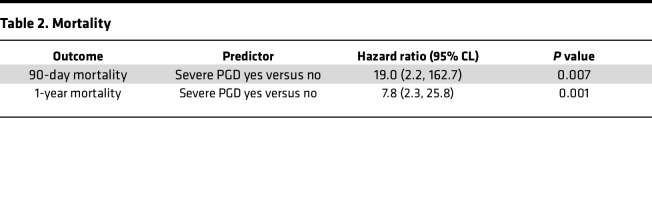
Mortality

## Version 1 11/08/2022

In-Press Preview

## Version 2 12/22/2022

Electronic publication
