# Supplementary material for: NKG2D receptor activation drives primary graft dysfunction severity and poor lung transplantation outcomes
Source: JCI Insight. 2022 Dec 22;7(24):e164603. doi: 10.1172/jci.insight.164603 (PMC9869973; doi:10.1172/jci.insight.164603)
Supplement: Supplemental data [file jciinsight-7-164603-s120.pdf]

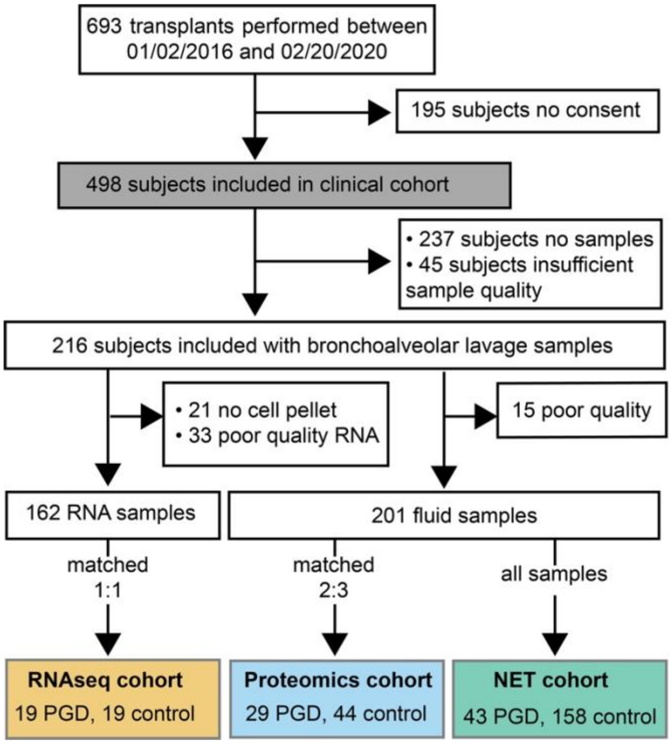

Supplemental Figure 1. Flow diagram of cohort.

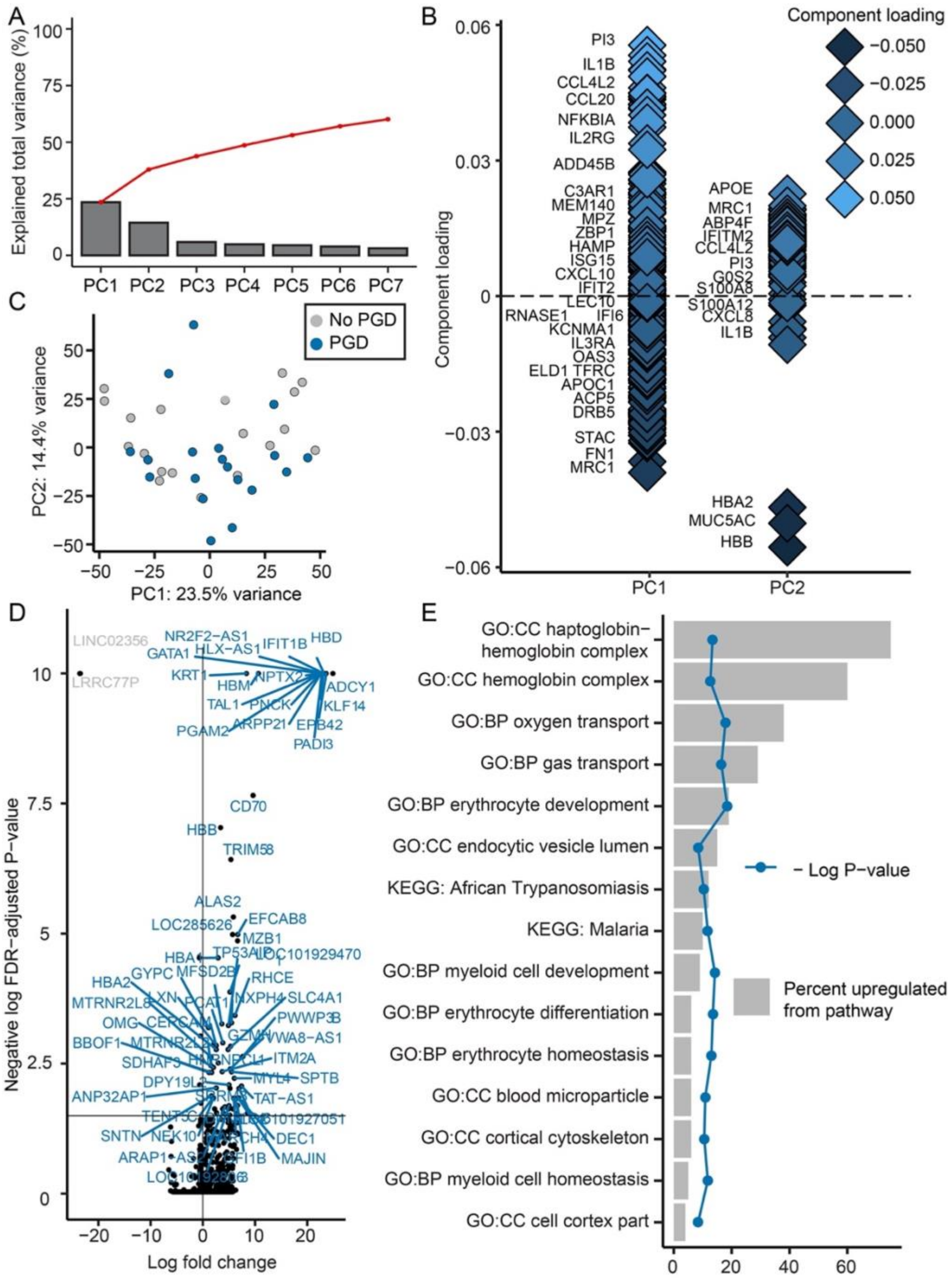

Supplemental Figure 2. Unsupervised BAL RNA sequencing analysis. RNA sequencing was performed on BAL from 38 participants on the first day after lung transplantation (severe PGD, n = 19). (**A**) Scree plot demonstrating that the first 7 principal components describe 65% of the variation in the genomic data set. (**B**) Principal component 1 and principal component 2 differentiate between severe PGD and no PGD. (**C**) Component loading plot illustrating the magnitude and vector of genes which constitute principal components 1 and 2. (**D**) Volcano plot of differentially expressed genes between cases and controls. E) Pathway analysis of differentially expressed genes.

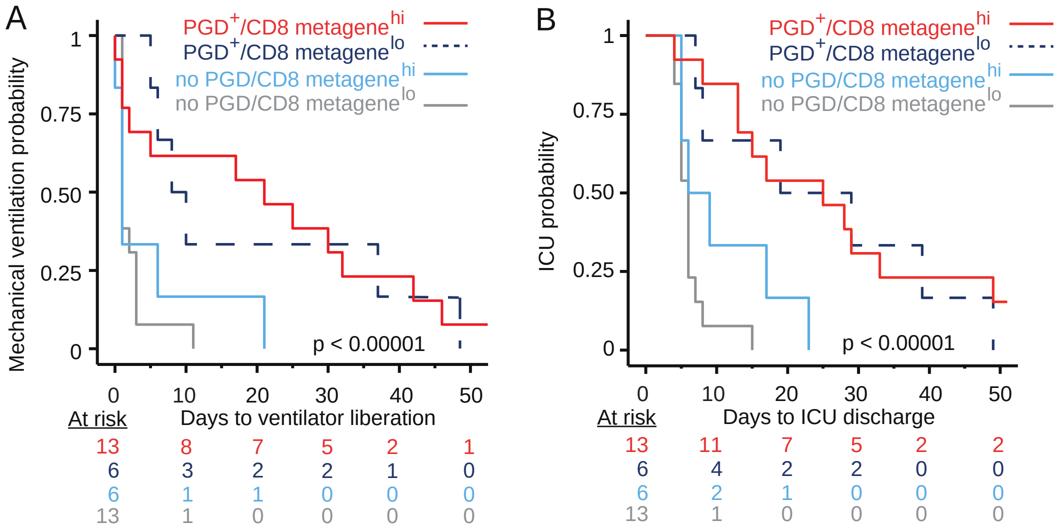

Supplemental Figure 3. CD8 T cell metagenes and ICU outcomes. A BAL CD8 T cell gene score was generated from RNA sequencing obtained from 38 participants on the first day after lung transplantation (severe PGD, n = 19). (**A**) Kaplan-Meier plot of mechanical ventilation time stratified by CD8 T cell gene scores and PGD. CD8 T cell gene score was not associated with increased risk for prolonged mechanical ventilation (HR 0.9, 95% CI 0.45-1.78, p = 0.77). (**B**) Kaplan-Meier plot of ICU length of stay stratified by CD8 T cell gene scores and PGD. Similarly, CD8 T cell gene score was not associated with increased risk ICU (HR 0.6, 95% CI 0.3 -1.2, p = 0.8). P values represent log-rank test for Kaplan-Meier plots of mechanical ventilation (**A**) and ICU survival times (**B**).

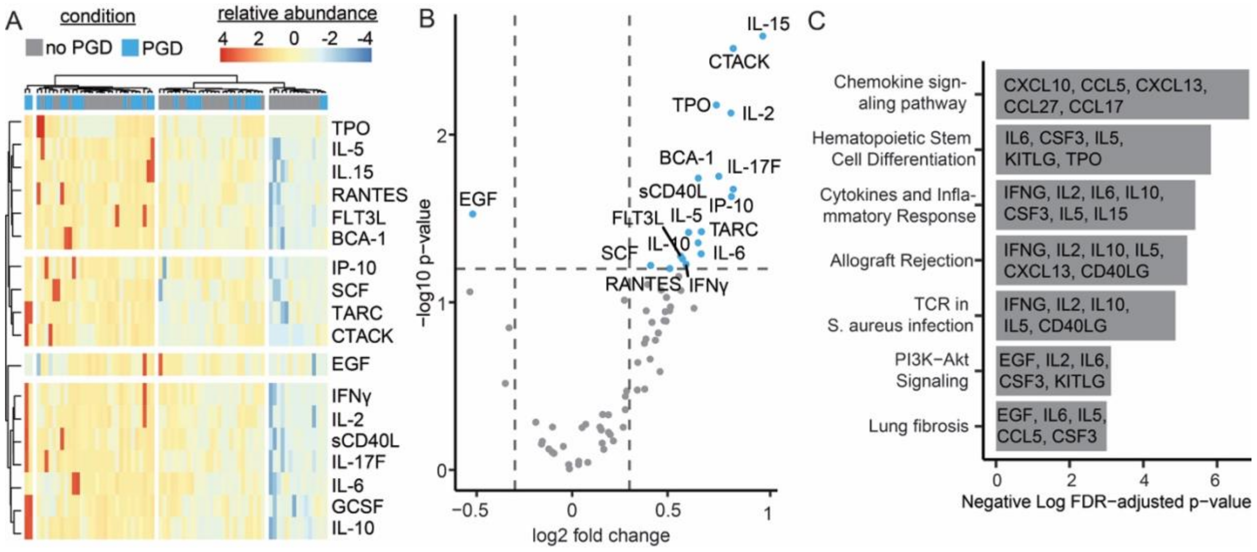

Supplemental Figure 4. Unsupervised proteomics analysis. BAL from 29 participants with severe PGD and 45 participants without PGD was assayed for 6 NK cell-related cytokines and 6 NKG2D receptor stress molecules on post-operative day 1 after lung transplantation. (**A**) A heatmap shows relative abundance of the top 25% differentially expressed proteins among the cohort. (**B**) Volcano plot of protein expression with annotations for proteins with negative log FDR-adjusted p-value < 1.2 and absolute log fold change > 0.3. (**C**) Pathway analysis with observed component proteins for each pathway.

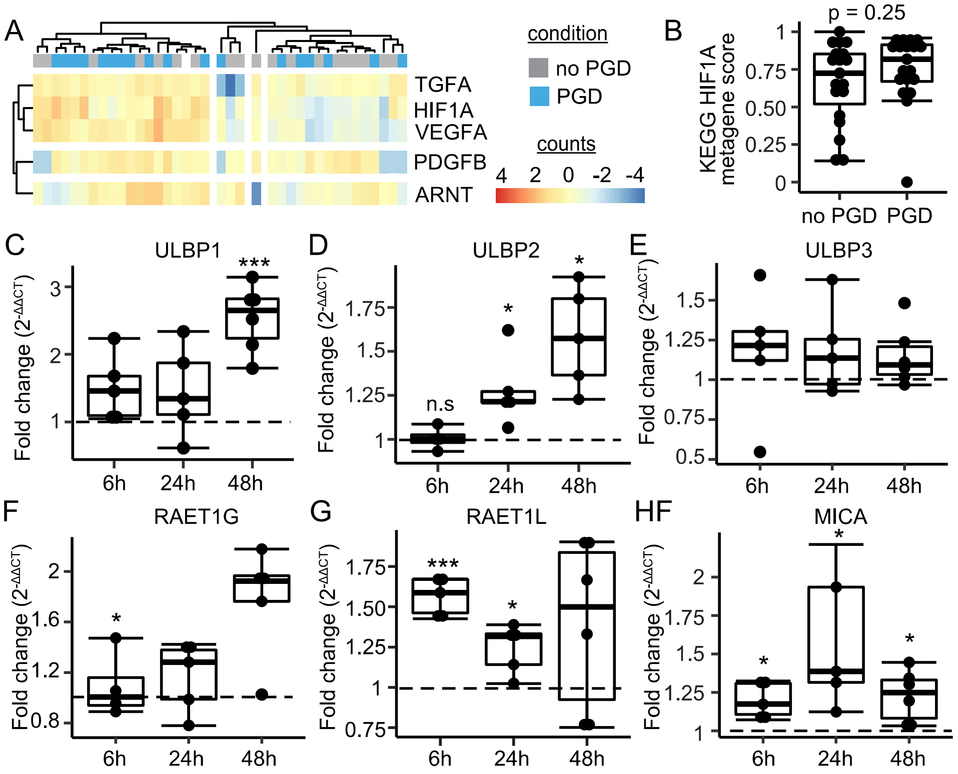

Supplemental Figure 5. Airway epithelial cell NKG2D ligand PCR. (A) A heatmap of KEGG HIF1A pathway gene transcripts are shown from BAL for participants with severe PGD (n = 19) and those without severe PGD (n = 19). (B) Metagene score for cumulative KEGG HIF1A pathway gene transcripts. Airway epithelial cells were exposed to 6 hours, 24 hours, or 48 hours of normoxia or hypoxia. mRNA was measured by PCR for (**C**) ULBP1, (**D**) ULBP2, (**E**) ULBP3, (**F**) RAET1G, (**G**) RAET1L, and (**H**) MICA. Summary data are displayed with box and whisker plots illustrating individual data points, bounded by boxes at 25th and 75th percentiles, and with medians depicted with bisecting lines. Individual P values are shown or represented by *$P < 0.05$, ** $P < 0.01$, *** $P < 0.001$, and differences assessed with Mann Whitney U test.

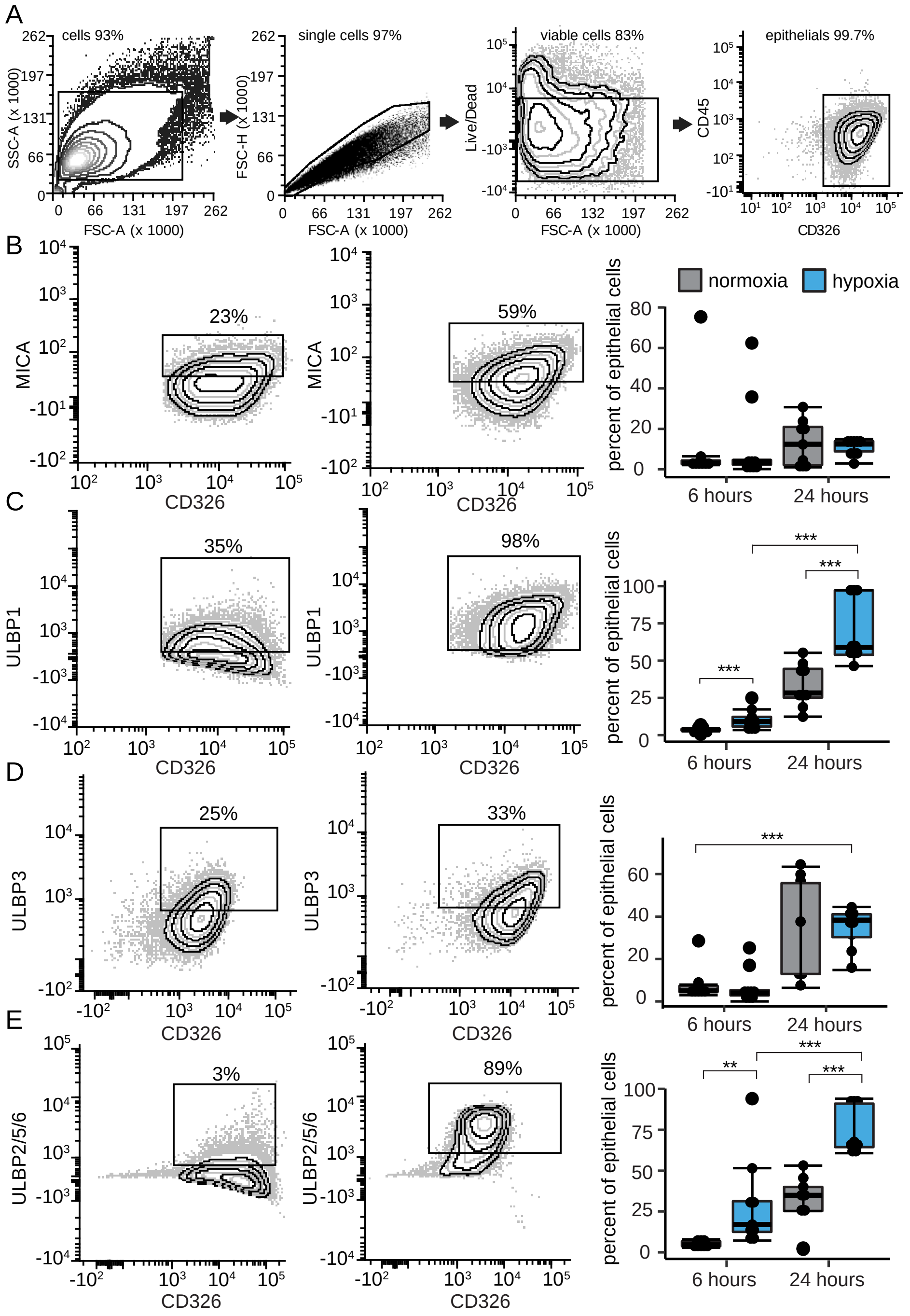

Supplemental Figure 6. Airway epithelial flow cytometry. Airway cells were exposed to 6 hours or 24 hours of normoxia or hypoxia and collected for flow cytometry. (**A**) The gating strategy for identification of airway epithelial cells. Representative contour plots of normoxia or hypoxia and summary data are displayed for NKG2D stress ligands; (**B**) MICA, (**C**) ULBP1, (**D**) ULBP3, and (**E**) ULPB2,5,6. Box and whisker plots depict individual data points, bounded by boxes at 25th and 75th percentiles, and with medians shown as bisecting lines. P values were calculated with the Mann Whitney U test and are represented by *P < 0.05, ** P < 0.01, *** P < 0.001.

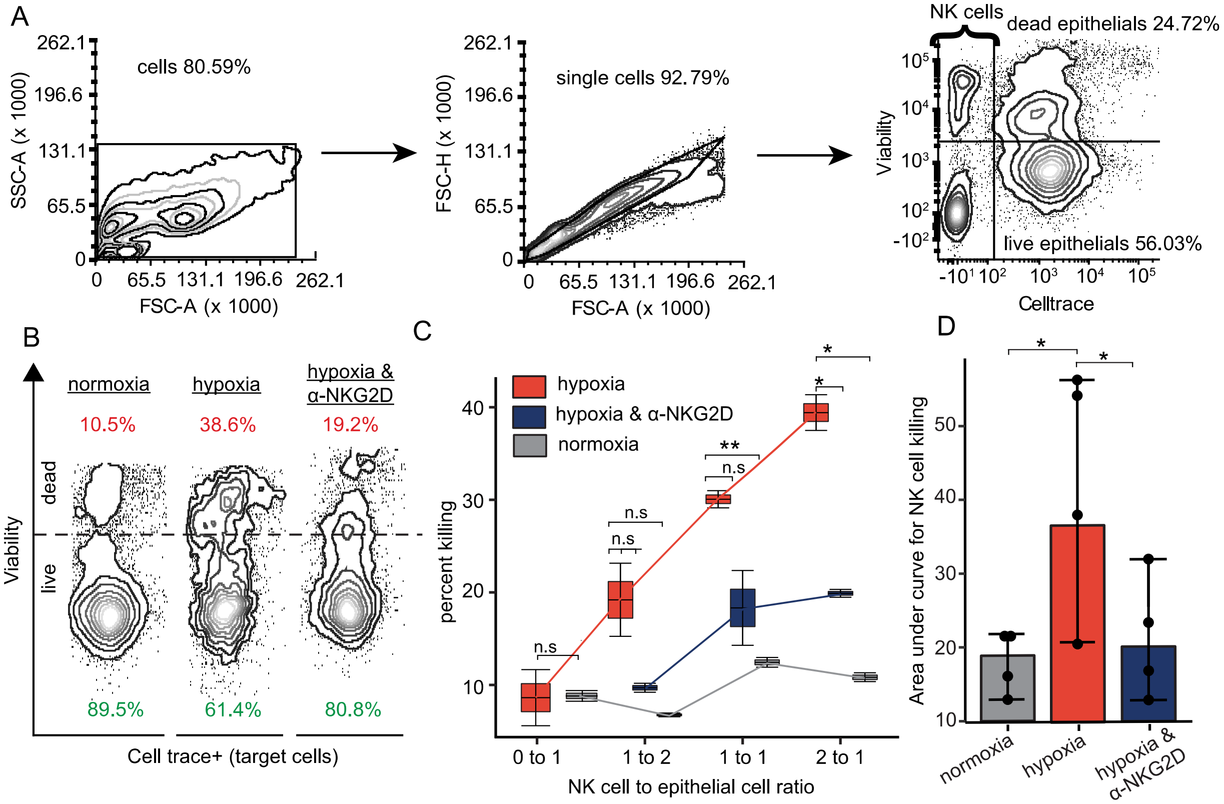

Supplemental Figure 7. NK cell cytotoxicity panel. (**A**) cells were identified, doublets were excluded, and epithelial cells were identified by the presence of cell trace and live cells were noted by exclusion of viability dye to assess NK cell killing of airway epithelial cells, hypoxic or normoxic control cells were co-cultured for 24 hours with primary human NK cells. **(B)** representative flow cytometry contour plots demonstrating NK cell killing by uptake of viability exclusion dye during normoxia, hypoxia, or hypoxia with NK cells treated with blocking NKG2D antibody. (**C**) Airway epithelial cell killing is plotted across several NK cell to epithelial ratios. (**D**) Area under the curve for cytotoxicity was assessed for the 3 conditions. Data are shown as box and whisker plots. Individual P values calculated with Mann Whitney U test. *P* values are represented by: *P <0.05, ** P <0.01.

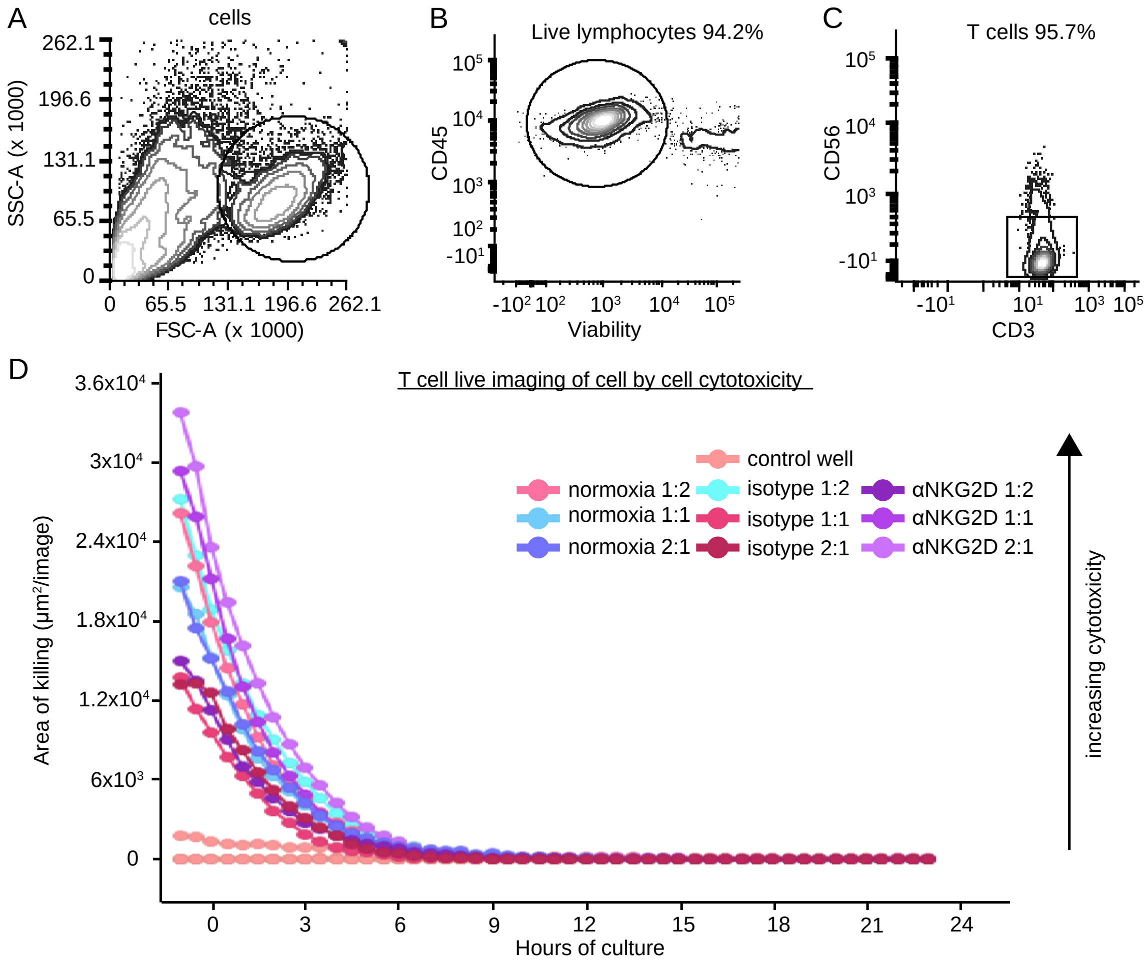

Supplemental Figure 8. T cell cytotoxicity of hypoxic airway epithelial cells. T cells were isolated from healthy donors and grown in culture. Flow cytometry identified the population used in the experiments as (**A**) singlet cells **(B)** viable lymphocytes (**C**) T cells with approaching 96% purity. (**D**) T cells were co-cultured with hypoxic or normoxic airway epithelial cells for 24 hours. T cells co-cultured with hypoxic airway epithelial cells were pre-treated with blocking NKG2D monoclonal antibody, or an isotype control antibody. Killing assay is shown as a cell-by-cell analysis representing total area per image of effector cell and cell death dye. There were no differences between these 3 groups, but more killing compared to the control group represented as target cells alone.

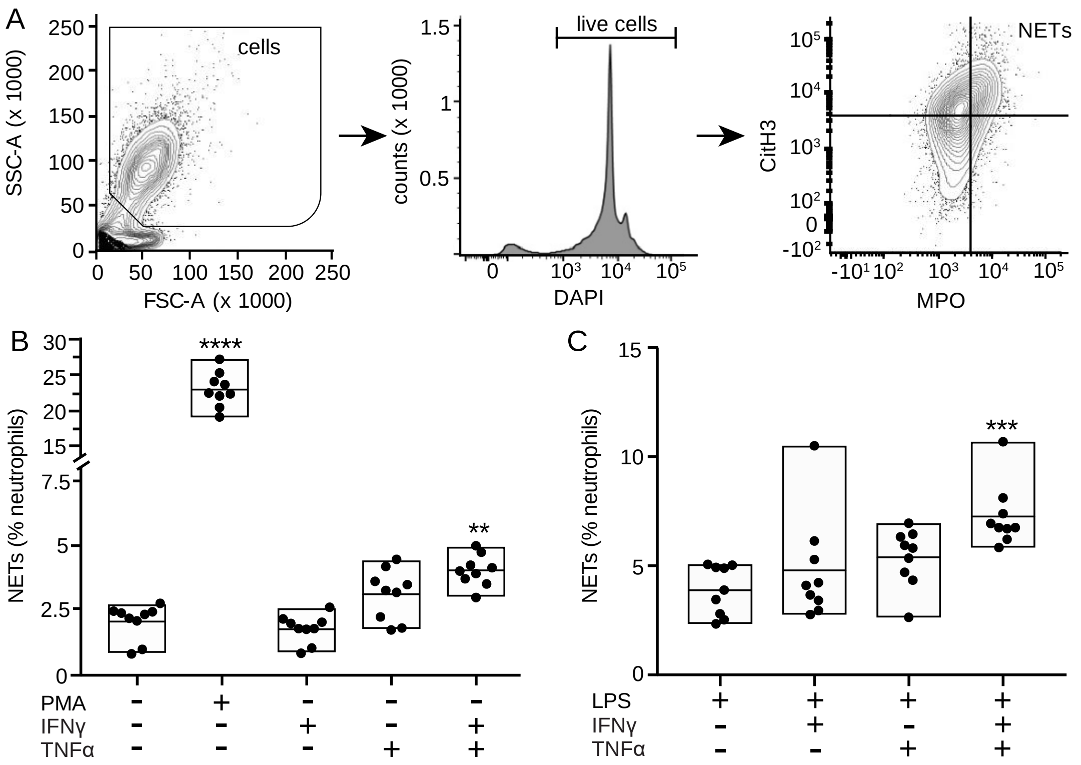

Supplemental Figure 9. Neutrophil extracellular trap flow cytometry. (**A**) NETs were gated from live cells and defined as MPO and CitH3 positive. (**B**) NETs as a percent of total neutrophils were measured under stimulation from PMA (positive control), normal saline (negative control), IFNγ, TNFα, or both cytokines and compared to negative control. (**C**) NETs as a percent of total neutrophils were also measured with priming by IFNγ, TNFα or both cytokines followed by stimulation with LPS and compared to LPS alone. Summary data are displayed with box and whisker plots illustrating individual data points, bound by boxes at 25th and 75th percentiles, and with medians depicted with bisecting lines. P values were calculated with the Mann Whitney U test and Benjamini-Hochberg correction for multiple comparisons and represented by *$P < 0.05$, ** $P < 0.01$, *** $P < 0.001$, **** $P < 0.0001$.

SUPPLEMENTAL TABLES

Supplemental Table 1. Univariate Donor and Recipient Characteristics

**Characteristics**	Total (N = 498)
**Donor variables**	
Donor age, mean ± SD	38.0 ± 14.8
Donor, Male, n (%)	334 (67.1)
Donor smoking > 20 pack-years, n (%)	29 (5.9)
**Recipient variables**	
Severe PGD, n (%)	105 (21.1)
Age at transplant, mean ± SD (per decade)	57.9 ± 12.4
Male, n (%)	302 (60.8)
Ischemic time, hours, mean ± SD	7.0 ± 3.3
Bilateral transplant, n (%)	389 (78.1)
Intraoperative cardiopulmonary bypass, n (%)	348 (69.9)
BMI, mean ± SD	25.5 ± 4.6
BMI Categories, n (%)	
<18.5	31 (6.2)
18.5~25	193 (38.8)
25~30	192 (38.6)
>=30	82 (16.5)
Diagnosis, n (%)	
COPD	52 (10.4)
PAH	26 (5.2)
CF	37 (7.4)
IPF	193 (38.8)
Non IPF ILD	190 (38.2)
Pulmonary Arterial Systolic Pressure, mmHg, mean ± SD	42.4 ± 18.1
LAS at transplant, mean ± SD	53.9 ± 19.0

Supplemental Table 2. PGD grade, N (%)

Day	PGD 0/1	PGD 2	PGD 3
0	223 (48.3)	116 (25.1)	123 (26.6)
1	258 (55.8)	117 (25.3)	87(18.8)
2	263 (55.0)	130 (27.2)	85 (17.8)
3	304 (63.2)	110 (22.9)	67 (13.9)

Supplemental Table 3. RNA cohort characteristics

	no PGD	PGD	P value
Participants (n)	19	19	
Age at transplant, mean ± SD (years)	62.1 ± 9.3	56.3 ± 15.8	0.18
Male sex (%)	10 (52.6)	6 (31.6)	0.32
Transplant type: N (%)			
Double	19 (100)	18 (94.8)	1
Heart and Lung	-	-	
Single	-	1 (5.2)	
Race/Ethnicity: N (%)			0.94
Caucasian	11 (57.9)	10 (52.6)	
African American	2 (10.5)	2 (10.5)	
Hispanic	-	-	
Other	6 (31.6)	7 (36.8)	
Indication: N (%)			0.96
A (COPD)	3 (15.8)	2 (10.5)	
B (Pulmonary Hypertension)	1 (5.2)	1 (5.2)	
C (CF)	1 (5.2)	1 (5.2)	
D (Pulmonary Fibrosis)	14 (73.7)	15 (78.9)	

Supplemental Table 4. Proteomics cohort characteristics

	no PGD	PGD	P value
Participants (n)	44	29	
Age at transplant, mean ± SD (years)	58.8 ± 12.9	58.1 ± 13.6	0.82
Male sex (%)	30 (68.2)	12 (41.4)	0.04
Transplant type: N (%)			
Double	41 (93.2)	26 (89.7)	0.92
Heart and Lung	-	-	
Single	3 (6.8)	3 (10.3)	
Race/Ethnicity: N (%)			0.66
Caucasian	34 (77.3)	20 (69)	
African American	2 (4.5)	2 (6.9)	
Hispanic	1 (2.3)	-	
Other	7 (15.9)	7 (24.1)	
Indication: N (%)			0.2
A (COPD)	8 (18.2)	2 (6.9)	
B (Pulmonary Hypertension)	2 (4.5)	2 (6.9)	
C (CF)	6 (3.6)	1 (3.4)	
D (Pulmonary Fibrosis)	28 (63.6)	24 (82.8)	

Supplemental Table 5. NET cohort characteristics

	no PGD	PGD	P value
Participants (n)	158	43	
Age at transplant, mean ± SD (years)	59 ± 11.8	57.1 ± 14.2	0.38
Male sex (%)	102 (63.3)	22 (51.2)	0.15
Transplant type: N (%)			
Double	135 (85.4)	40 (93)	0.29
Heart and Lung	-	-	
Single	23 (14.6)	3 (7)	
Race/Ethnicity: N (%)			0.69
Caucasian	100 (63.3)	27 (62.8)	
African American	7 (4.4)	1 (2.3)	
Hispanic	13 (8.2)	2 (4.7)	
Other	38 (24.1)	13 (30.2)	
Indication: N (%)			0.02
A (COPD)	23 (14.6)	2 (4.7)	
B (Pulmonary Hypertension)	3 (1.9)	3 (7)	
C (CF)	12 (7.6)	-	
D (Pulmonary Fibrosis)	120 (75.9)	38 (888.4)	

Supplemental Table 6. Genes comprising metagenes

Metagene Score	Genes
B cells	*ABCB4, ADAM28, BACH2, BANK1, BCL7A, BEND5, BLK, BRAF, CD180, CD19, CD1C,CD22, CD37, CD69, CD72, CD79A, CD79B, CR2, CXCR5, EAF2, FAIM3, FCER2,FCGR2B, FCRL2, FRK, GPR18, GUSBP11, HHEX, HLA-DOB, IGHD, IGHM, IGKC, IGLL3P, IL4R, IRF8, KIAA0226L, LINC00921, LTB, LY86, MEP1A, MICAL3, MS4A1, NIPSNAP3B, NMBR, P2RX5, P2RY14, PNOC, PSG2, PTPRCAP, RALGPS2, RASGRP2, SELL, SIK1, SLC12A1, SPIB, STAP1, TCL1A, UGT1A8, VPREB3, ZNF286A*
CD4 T cells	*ACAP1,ANKRD55, ATHL1, BCL11B, CCR7, CD2, CD247, CD27, CD3D, CD3G, CD40LG,CD7, CXorf57, DPP4, DSC1, EPHA1, FAIM3, FLJ13197, FLT3LG, GAL3ST4, GALR1,GPR1, GRAP2, GZMM, ICOS, IL7R, ITK, LAT, LCK, LEF1, LIME1, LTB, LY9, MAP4K1, MAP4K2, MAP9, RASGRP2, RPL3P7, SERGEF, SH2D1A, SIRPG, TCF7, TRAC, TRAT1, TRAV13-1, TRBC1, UBASH3A, VILL, WNT7A, ZAP70, ZNF204P, ZNF324*
CD8 T cells	*BCL11B, CCL5,CD2, CD247, CD27, CD3D, CD3E, CD3G, CD6, CD69, CD7, CD8A, CD8B, CD96, CRTAM, CST7, CTSW, DPP4, DSC1, DUSP2, FAIM3,FLT3LG, GNLY, GPR171, GRAP2, ICOS, IGKC, IL7R, ITK, LAG3, LCK, LEF1, LIME1, LTB, LY9, MAP4K1, MAP9, PIK3IP1, PRF1, PTGDR, PTPRCAP, PVRIG, RASA3, RPL3P7, SH2D1A, SIRPG, TCF7, TRAC, TRAT1, TRAV12-2, TRAV13-1, TRBC1, TRDC, UBASH3A, ZAP70*
NK Cells	*XCL1, GZMH, EOMES, KLRD1, IL15RA, FCGR3A , IL2RB, GZMB, KLRC1, IFNG*
Monocytes	*AIF1, APOBEC3A, AQP9, ASGR1, ASGR2, BST1, C5AR1, CCR2, CD1D, CD33, CD68, CDA, CFP, CHST15, CLEC4A, CLEC7A, REB5, CSF3R, FAM198B, FCN1, FES, FOSB, FPR1, FZD2, HCK, HK3, NMT, HPSE, IGSF6, LILRA2, LILRA3, LILRB2, LST1, MEFV, MNDA, MS4A6A, NCF2, NFE2, NLRP3, NOD2, P2RY13, PADI4, RNASE2, RNASE6, S100A12, SLC15A3, TLR2, TLR7, TLR8, UPK3A, VNN1, VNN2*
Macrophages	*LAG3, LAMP3,LILRA3, LILRB2, NOD2, PLA1A, PTGIR, RASSF4, RSAD2, SIGLEC1, SLAMF1, SLC15A3, SLC2A6, SOCS1, TLR7, TLR8, TNFAIP6, TNIP3, TRPM4*
Mast Cells	*ATP8B4, BPI, CCL1, CCL20, CCL4, CD33, CLC, CMA1, CPA3, CSF2, CTSG, CXCL3, FCER1A, GZMB, HDC, HOXA1, HPGDS, IL18R1, IL1A, IL1B, IL1RL1, IL3, IL5, LINC00597, MARCH3, MS4A2, MS4A3, MYB, NOX3, NTRK1, P2RX1, PRG2, RAB27B, RGS13,SLC12A8, STXBP6, TEC, TPSAB1*
Neutrophil Extracellular Traps	*MPO, FOXO3, GNLY, NOX4, TLR7, TLR8, TLR9, FCRG1A, AIM2, FCGR3B, ITGB2, ICAM3, GAPDH, TPI1, LDHB, DLAT, HK1, HK2, HIST1H1B, DNASE1, DNASE2, DNASE2B, HMGB1, LDHAL6B, ALDH7A1*
NK Cell and Stress Ligand Score	*XCL1, GZMH, EOMES, KLRD1, IL15RA, FCGR3A, IL2RB, GZMB, KLRC1, IFNG, MICA, MICB, ULBP1, ULBP2, ULBP3, RAET1E, RAET1G, RAET1L*
